# Enneagram in EM

**DOI:** 10.21980/J8ZM0G

**Published:** 2023-10-31

**Authors:** Megan Cifuni, Cami Pfennig, Caroline Astemborski

**Affiliations:** * Prisma Health Upstate, Department of Emergency Medicine, Greenville, SC

## Abstract

**Audience:**

This is a lecture paired with facilitated small group sessions and is targeted towards emergency medicine residents and physicians.

**Background:**

The enneagram is a well-established and popular personality theory that asserts that there are nine basic personality types, and that each enneagram type, 1–9, operates from a basic fear and a basic desire that produces predictable behavioral patterns and preferences.^1^–^2^ The enneagram has long been used as a tool to enhance self-awareness and to better understand internal defenses and reactions,^3^–^5^ and as such, it has been increasingly utilized to enhance self-growth and development in the fields of education, parenting, and business.^6^–^7^ While some studies have used the enneagram as a tool to predict natural empathy or stress levels of those in the medical field, particularly in nursing and medical school students,^8^–^9^ little has been published on the use of the enneagram as a tool to enhance self-awareness, leadership, and teamwork in the medical field. Emergency medicine is a specialty in which residents and physicians must not only be self-aware but must also be attuned to the dynamics of their healthcare team in order to succeed. We believe that the enneagram is the ideal tool to enhance these crucial skills.

**Educational Objectives:**

The primary aim of this session was to enhance participants’ self-awareness by identifying their enneagram type and therefore their predictable behavioral patterns. The secondary aim was to discuss strategies to improve teamwork and physician team leadership by directly addressing the type’s strengths and weaknesses in these interactions.

By the end of this session, the learner will be able to: 1) Self-identify with a primary enneagram personality type. 2) List the fears, desires, and motivations of the enneagram type. 3) Describe struggles in interacting with other disparate enneagram types. 4) Discuss strategies for success in facing conflict and interacting with other team members.

**Educational Methods:**

This lecture was designed to educate emergency department physicians and residents on the enneagram tool. The introductory lecture takes about 20 minutes, and following this foundational presentation, learners split into small groups. Small group sessions take an additional 20 minutes during which facilitators guide learners through a discussion on their enneagram type and the potential strengths and challenges that each type might face in professional situations. This session was hosted during an Emergency Medicine Resident Education Conference. Due to COVID-19 restrictions, the session was presented virtually on a synchronous video platform with small group breakout rooms.

**Research Methods:**

Following the session, the educational content was evaluated by our residents and faculty by a Likert reaction survey. The survey assessed both the form and effectiveness of the delivery method and the impact of the content in the session.

**Results:**

A total of 17 responses with a mix of faculty and PGY years were collected after the session. In the post-session survey, 23% (6) of participants reported that the session was “moderately important in better understanding myself,” and 38.5% (4) of participants reported that the session was “quite important in better understanding myself.” 62% (11) of participants agreed or strongly agreed that the session helped them to understand their peers’ personalities and communication preferences.

**Discussion:**

Overall, this educational content and delivery in this format was well received and effective in enhancing residents’ understanding of themselves and their team’s personalities. Our residents and faculty engaged in insightful conversations around their own enneagram type and shared their successes and struggles in interacting with other enneagram types. At the end of the session, our participants left with not only greater self-awareness but also with an appreciation for the preferences and personalities of others on the medical team.

**Topics:**

Enneagram, leadership, teamwork, self-awareness, emotional intelligence.

## USER GUIDE

[Table t1-jetem-8-4-l1]

**Table t1-jetem-8-4-l1:** 

List of Resources:	
Abstract	1
User Guide	4
Appendix 1: Enneagram in EM Lecture PowerPoint	6
Appendix 2: Enneagram in EM Presenter Notes	7
Appendix 3: Enneagram Subtypes	18
Appendix 4: Discussion Questions for Small Groups	21
Appendix 5: Enneagram in EM Session Evaluation	22

**Learner Audience:**

Interns, Junior Residents, Senior Residents, Attending Physicians

**Time Required for Implementation:** 40 minutes

**Recommended Number of Learners per Instructor:**

Ideal for 20–50 learners total. 10 Small groups would then contain ~5 learners. Each small group would need a facilitator.

**Topics:**

Enneagram, leadership, teamwork, self-awareness, emotional intelligence.

**Objectives:**

By the end of this session, the learner will be able to:

1. Self-identify with a primary enneagram personality type.
2. List the fears, desires, and motivations of the enneagram type.
3. Describe struggles in interacting with other disparate enneagram types.
4. Discuss strategies for success in facing conflict and interacting with other team members.

### Linked objectives and methods

Learners will self-identify with a primary enneagram personality type (objective 1), by taking and completing the included online enneagram personality test. During the session, the PowerPoint presentation (large group presentation), will outline the fears, desires, and motivations of the enneagram type (objective 2). In small groups, facilitators will lead discussions around the struggles of interacting with other disparate enneagram types (objective 3), and will discuss strategies for success in facing conflict and interacting with other team members (objective 4).

### Recommended pre-reading for instructor

Prereading recommended for lecturer and small group facilitators:

- Take the enneagram test to understand your own type: https://www.truity.com/test/enneagram-personality-test.
  ○ The Enneagram Personality Test (2021). *Explore the nine types and discover who you truly are.* Retrieved June 15, 2023 from: https://www.truity.com/test/enneagram-personality-test
  ○ The website provides you with your enneagram subtype without cost. For an in-depth description of your subtype, the website will ask you to pay for additional information. To explore subtypes without a cost see the included Word^®^ document with subtype descriptions.
- Included enneagram subtype descriptions, Word^®^ document.
  ○ Further explore your enneagram subtype using the attached Word^®^ document to learn more about your subtype.
- Descriptions of each enneagram type: https://www.enneagraminstitute.com/type-descriptions
  ○ The Enneagram Institute. (2022). *Type descriptions*. The Enneagram Institute. Retrieved September 28, 2022, from https://www.enneagraminstitute.com/type-descriptions
- Analysis of reach enneagram type in the workplace: https://www.success.com/the-enneagram-at-work/
  ○ Paulk, S. (2022, April 2). *The enneagram at work: Which number are you?* SUCCESS. Retrieved September 28, 2022, from https://www.success.com/the-enneagram-at-work/
- Discussion on enneagram types and conflict resolution: https://www.collaborativepractice.com/system/files/The%20Enneagram%20types%20dealing%20with%20Conflict-%20IACP.pdf
  ○ Sneh Lurie, G. S. (2019, October 3). *The Enneagram Types Dealing with Conflict*. Collaborative Practice. Retrieved September 28, 2022, from https://www.collaborativepractice.com/system/files/The%20Enneagram%20types%20dealing%20with%20Conflict-%20IACP.pdf
- Optional addition resource: *The Essential Enneagram* by David Daniels, M.D. and Virginia Price Ph.D.
  ○ Daniels, D. N., & Price, V. A. (2009). *The essential enneagram: The definitive personality test and self-discovery guide*. HarperOne.

### Learner responsible content (LRC)

Before the session, participants should take the enneagram test to identify their enneagram type.

- Take the enneagram test to understand your own type: https://www.truity.com/test/enneagram-personality-test
  ○ The Enneagram Personality Test (2021). *Explore the nine types and discover who you truly are.* Retrieved June 15, 2023 from: https://www.truity.com/test/enneagram-personality-test
- The test is offered for free, but you will be asked to pay to get detailed analysis of your subtype. Do not pay for a subscription to explore your subtype. We will explore your subtype during the session.

### Results and Tips for Successful Implementation

The initial foundational understanding of the enneagram and the motivations of each type is best conveyed in a large group setting, most efficiently through a single presenter and PowerPoint. We found that by dividing subsequently into smaller groups for facilitated discussion, our learners could become active participants, sharing their own experiences, struggles and potential solutions.

We presented this session during our emergency medicine residency education conference. Following the conference, we surveyed the participants using a Likert reaction survey to solicit their feedback on the method of delivery and content. In the post-session survey, 23% (6) of participants reported that the session was “moderately important in better understanding myself,” and 38.5% (4) of participants reported that the session was “quite important in better understanding myself.” 62% (11) of participants agreed or strongly agreed that the session helped them to understand their peers’ personalities and communication preferences.

Unfortunately, this session was initially held during a COVID surge, requiring us to gather via a synchronous online video platform, utilizing breakout rooms for small group sessions, and we suspect that an in-person session would result in an even greater report of improvement in understanding of communication preferences. While this session still received excellent verbal and written feedback via survey results, in-person sessions would be even more impactful.

### Technology necessary

Computer and projector.

### Associated content (optional)

The structure of the sessions is as follows:

- 15 minutes of pre-meeting preparation in taking the enneagram test.
- 20 minutes introduction to the enneagram in large group via PowerPoint presentation.
- 20 minutes of facilitated small group discussion. Groups are divided up based on self-identified enneagram type (1–9). Facilitators guide is also included.

### Technology necessary

A computer or cellular device is necessary to complete the course surveys.

### Associated Content

- Enneagram in EM Lecture PowerPoint
- Enneagram in EM Lecture Presenter Notes
- Enneagram Subtypes
- Discussion Questions for Small Groups
- Enneagram in EM Session Evaluation

## Supplementary Information



## Figures and Tables

**Figure f1-jetem-8-4-l1:**
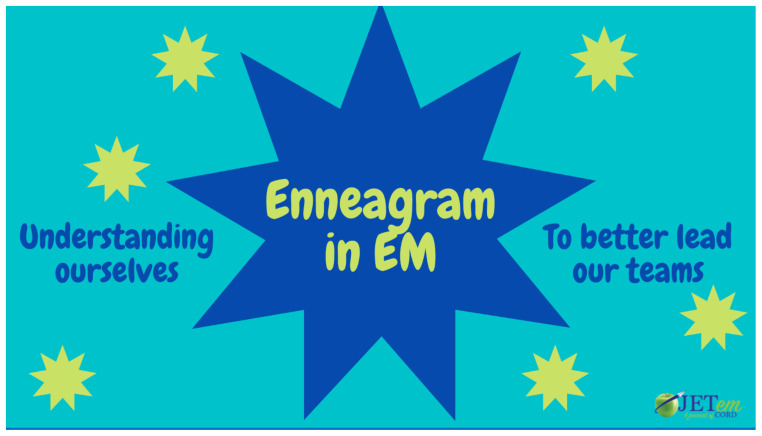
Please see associated PowerPoint file

## Additional reading and resources

- Hur Y, Lee K. Analysis of medical students’ enneagram personality types, stress, and developmental level. *Korean J Med Educ.* 2011;23 (3): 175–184. Publication Date (Web): 2011 September 30 (Original Article). At: doi:https://doi.org/10.3946/kjme.2011.23.3.175
- Descriptions of each enneagram type: https://www.enneagraminstitute.com/type-descriptions
- Analysis of each enneagram type in the workplace: https://www.success.com/the-enneagram-at-work/
- Discussion of enneagram types and conflict resolution: https://www.collaborativepractice.com/system/files/The%20Enneagram%20types%20dealing%20with%20Conflict-%20IACP.pdf
- Additional reading: Daniels D, Price V. *The Essential Enneagram.* HarperOne; Revised, Updated ed. November 4, 2009.

## USER GUIDE AND LEARNER MATERIALS

### Appendix 1 Emmeagram in EM Lecture PowerPoint

[Fig f1-jetem-8-4-l1]

### Appendix 2 Enneagram in EM Presenter Notes/Script

#### Slide 1

The enneagram is a personality theory that has been around for decades. In recent years it has grown in popularity in many aspects of life including parenting, athletics, and in the field of business as a tool to enhance emotional intelligence. The theory asserts that each person falls into one of nine basic personality types. These types each represent a basic fear and basic desire, and from this, we can derive predictable behavior patterns.

Hopefully, you have all taken the enneagram test assigned and have come to identify with an enneagram type (1–9).

#### Slide 2

Our primary goal here today is to understand OURSELVES better. By better understanding your OWN personality, you can lead and work with others in a more effective and efficient manner. In effect, we are building upon our own emotional intelligence.

So our study of the enneagram starts with self-understanding – what motivates you, what are you afraid of, and how do you intuitively deal with conflict and stressful situations?

#### Slide 3

With the tool of the enneagram, we can better understand how and why others see the world differently from us. This awareness leads to greater compassion and acceptance of others.

As a physician leader, you interact with many different team members, most of whom probably see the world differently than you do. Different perspectives, values, and preferences sometimes can lead to misunderstanding and frustration. But with the enneagram, we can better understand how and why others see the world differently from us. This awareness leads to greater compassion and acceptance of others. We can apply this knowledge to adapt our approach to various team members.

#### Slide 4

But understanding our own personalities can also help us understand our own behavior (and perhaps challenge it) in moments of stress. We all have our own individual quirks, flaws, and shortcomings. And the behaviors may not exist just because we’re stubborn, wrong-headed or lack willpower. It’s just the way we’re wired.

#### Slide 5

There will always be people on our teams that make us a little crazy.

Even when you don’t know what someone else’s type is, just being aware of the fundamentally different perspectives and attitudes of each type can be eye-opening. That awareness makes it a lot easier to cut others some slack. Our differences don’t always have to be frustrating or divisive. They can be a source of humor and even a way to connect.

#### Slide 6

Have you been interacting with difficult team members or approaching difficult situations in the SAME way, over and over again? And are you frustrated that you don’t get different results?

The Enneagram explains how we get into our particular ruts of behaving, thinking, and feeling, and then offers ways that we can challenge those behavioral patterns when needed.

Knowing your enneagram type is extremely helpful—maybe even essential—if you are in the process of attempting to change your behavior.

#### Slide 7

On a personal level, the enneagram can help you deepen your relationship with your partner or develop clearer communication with friends and family members. At work, it can help you get along better with your co-workers, understand your boss, and become more effective. What you take from it depends on how you come at it and what it is you’re looking for.

#### Slide 8

Now, let’s talks through the different enneagram types.

The Type One physician is concerned with goodness, perfection, and integrity.

They may believe that it is their responsibility to point out the flaws in their workplace, the medical profession, their patients, their own work, and pretty much everyone and everything else around them, but most especially themselves.

You may hear a Type One say:

- “Can’t you do better than this?”
- “Why can’t any of the specialty colleagues do things right?!”
- “That wasn’t good enough; I’m going to do it over again to get it right.”

They may constantly point out mistakes and be critical of both others and of themselves.

On the other hand, a healthy and growing type one physician will increasingly exemplify integrity and appreciate the inherent goodness in themselves and others, even in the presence of flaws.

https://www.thescopeofpractice.com/how-physicians-can-use-the-enneagram-as-a-tool-for-personal-and-professional-development/

#### Slide 9

You can support Type Ones by helping them learn to pace themselves.

Show them they are valued not just for what they do, but who they are.

Show them that anger, openness, and honesty are ok, and that they are allowed to relax and be spontaneous.

https://compassionatelearning.org/enneagram-personalities-students-copingstrategies-emotionalintelligence-compassion/enneagram-personalities-students-resilience-emotionalintelligence-typeone-student/

#### Slide 10

Type Ones have high standards for work, including documentation. If charting is taking an inordinate amount of time, do a trial period of more succinct notes for two weeks. You can always go back to the longer notes, but perhaps shorter notes (with even a few typos) may be acceptable.

https://rendia.com/resources/insights/how-enneagram-can-improve-management-style/

Type Ones have a very strong inner critic. See how it feels to take on a kinder, more forgiving tone with yourself. There is often a subconscious fear that if Type Ones give themselves a little slack, they will turn into slackers,

#### Slide 11

Type Twos are the people-pleasing mentors.

They are concerned with attending to others’ needs.

They are the ones always available to serve and care for their patients, coworkers, bosses, family, friends, and even strangers.

You may hear Twos say:

- “Oh, it was not a problem at all! I’ll be there for you anytime.”
- “There’s no need to thank me. It was absolutely my pleasure. Really.”
- “Go home. I’ll take care of it.”

However, this can happen at the expense of their own needs. As a result, they may unknowingly harbor the desire to get the very attention they are giving others. On the other hand, a healthy and growing Type Two physician will increasingly exemplify generosity while still being able to relate to others in a mutually beneficial way.

https://www.thescopeofpractice.com/how-physicians-can-use-the-enneagram-as-a-tool-for-personal-and-professional-development/

#### Slide 12

Type Twos tend to work for approval. You may notice this in a drive to:

- seek good grades
- volunteer for projects or assignments
- very sensitive to explicit or implicit criticism; may even see disagreement as disapproval

https://compassionatelearning.org/enneagram-personalities-students-copingstrategies-emotionalintelligence-compassion/enneagram-typetwo-student/

#### Slide 13

Type Twos can be at high risk for compassion fatigue and burnout from giving too much.

If you are a Type Two, before saying “yes” to additional commitments, press the pause button and consider whether this obligation serves you. Is it something YOU want to do? Take stock of your self-care and personal time. Is all your time going towards work and family, with little left over for you? Try putting yourself first for a few weeks and see how that changes things. To do this, you will likely need to ask for more from others and redefine some boundaries.

https://doctorscrossing.com/burning-out-recommendations-for-your-personality-type/

#### Slide 14

Type Threes are the star of the class.

They may believe they will find their value by continuing to complete achievement after achievement and try even harder when they are not satisfied. Conversely, a healthy and growing Type Three physician will be driven towards absolutely grand goals and objectives while keeping in mind that they have worth and value that are not measured by their accomplishments.

https://www.thescopeofpractice.com/how-physicians-can-use-the-enneagram-as-a-tool-for-personal-and-professional-development/

#### Slide 15

Type Threes will be driven to excel. And you may find they:

- work quickly, may cut corners to get done faster
- need to produce and achieve all the time
- may brag/show off
- enjoy competition/contests
- sensitive to criticism
- keep quiet when not doing well
- turn failure into partial success
- avoid areas in which they know they won’t excel

https://compassionatelearning.org/enneagram-personalities-students-copingstrategies-emotionalintelligence-compassion/enneagram-typethree-student/

#### Slide 16

Being a driven Type Three can result in career success and a great CV, but it can also leave one feeling empty and disconnected from one’s heart. As an achiever type, they may have put your feelings aside to reach their goals. They should slow down in order to find out what is driving the achievement, and ask themselves if there is something else their hearts desire.

#### Slide 17

Type Fours are the Misunderstood Creative--very sensitive to criticism (see this as personal rejection).

https://compassionatelearning.org/enneagram-personalities-students-copingstrategies-emotionalintelligence-compassion/enneagram-typefour-student/

Type Fours want special, meaningful experiences.

They value self-expression, creativity, emotional authenticity.

The Type Four physician is concerned with their individual significance and importance. They are the ones constantly expressing their victimhood, talking about how they are not appreciated by patients, coworkers, and even friends and family. In addition, they seem to revel in all of this.

You may hear a Type Four saying:

- “Woe is me.”
- “Yeah, my job is terrible, and no one cares about me, but that’s who I am, and I’m going to be me.”

They create atmospheres or moods that highlight and validate their special situation and identity. Yet, healthy and growing Type Four physicians make use of their emotions and feelings to exemplify authenticity and creativity because they have a firm sense of their true significance.

https://www.thescopeofpractice.com/how-physicians-can-use-the-enneagram-as-a-tool-for-personal-and-professional-development/

#### Slide 18

Type Fours will create projects that are works of art.

You may find that type Fours:

- want their work to be extraordinary (not ordinary)
- may be artistically inclined
- turn boring work into something beautiful
- want special, meaningful experiences
- want each student’s uniqueness recognized
- don’t want to be compared with others

https://compassionatelearning.org/enneagram-personalities-students-copingstrategies-emotionalintelligence-compassion/enneagram-typefour-student/

#### Slide 19

Type Fours are highly creative, intuitive, and seek meaning and connection in the work. Because they like to express their ideas and unique approach, a work environment that is too confining and does not value their individuality will not be a good fit. Having a job primarily for income will not be sustainable.

https://doctorscrossing.com/burning-out-recommendations-for-your-personality-type/

#### Slide 20

Type Fives are the Intellectual Outsiders.

The Type Five physician is concerned with the ability to persevere and be in the world. They are the physicians who are always worried about being drained but also seeking more knowledge by getting more training, reading more, and attending more CME courses than required to feel more proficient in their work.

You may hear Type Fives say:

- “I’m not ready.”
- “I can’t go out today” (because they feel drained of energy and will be further drained by doing more).
- “Oh, I bought a book about how to do that recently” (but did not end up doing what the book taught them).

They may withdraw from work and professional scenarios and situations that seem draining to them. However, a healthy and growing Type Five physician can easily get into a flow or zone of innovative application of their knowledge and resources.

https://www.thescopeofpractice.com/how-physicians-can-use-the-enneagram-as-a-tool-for-personal-and-professional-development/

#### Slide 21

Type Fives have a strong ability to focus.

You may find that Type Fives:

- hate concentration being interrupted,
- prefer depth of knowledge to breadth of knowledge
- enjoy time alone and need time to think
- don’t like pressure of close supervision, thinking on their feet
- have active minds full of ideas and concepts
- feel safe “inside” and clumsy ‘“outside’”
- seek to reduce intrusion of their space

https://compassionatelearning.org/enneagram-personalities-students-copingstrategies-emotionalintelligence-compassion/enneagram-typefive-student/

#### Slide 22

Type Fives are innovators and deep thinkers. The ideal work setting is one where they can focus deeply without interruptions and work independently in their area(s) of interest. Diversifying patient care with research, teaching, and projects can be helpful in preventing burn out.

https://doctorscrossing.com/burning-out-recommendations-for-your-personality-type/

#### Slide 23

Type Sixes are the Questioning Friends.

The Type Six physician is concerned with trust and groundedness. They are the physicians overly concerned about the values and actions of their hospital or practice and make it a point to join or leave medical organizations based on if they know they can trust them and belong to that group.

You may hear type Sixes saying:

- “My specialty colleague has been suspicious lately; I don’t know if I can trust them.”
- “I’ll do what the group decides.”
- “Are you sure? I’m not sure. Are you?”

Their thoughts and trust can waver quickly and often, or, at the other extreme, barely budge at all. They can end up overly depending on others or even be antiauthoritarian simply for the sake of it. Alternatively, a healthy and growing Type Six physician brings togetherness to a group and fosters an atmosphere of stability of trust for themselves and others.

https://www.thescopeofpractice.com/how-physicians-can-use-the-enneagram-as-a-tool-for-personal-and-professional-development/

#### Slide 24

You may find that Type Sixes:

- question inconsistencies, assumptions
- seek to understand teacher’s experience, bias, preferences
- have uncertainty that breeds anxiety, worry
- want to observe first, get assumptions out of the way before participating
- over-questioning leads to analysis paralysis
- self-doubt leads to procrastination

https://compassionatelearning.org/enneagram-personalities-students-copingstrategies-emotionalintelligence-compassion/enneagram-typesix-student/

#### Slide 25

They excel in organizations due to hard work, problem-solving abilities, people skills, and desire to exceed expectations.

They should try to avoid spending unnecessary time second-guessing and asking other’s opinions. They should pay attention to how often they are worrying about the future.

#### Slide 26

Type Sevens are the Joyful Enthusiasts

The Type Seven physician is concerned with finding happiness by looking for something new. They are the physicians who can enjoy locum tenens work forever or who revel in hopping from one career to another (and sometimes back).

You may hear a Type Seven say:

- “I know I’ve only been here for six months, but I don’t think this is for me. See ya!”
- “I really enjoyed my week-long hiking vacation, so I am considering becoming a wilderness medicine specialist.”
- “Let’s try doing it in a new way. Maybe it will work better!”

They may be easily distracted and not able to settle into one place or project for long. In contrast, a healthy and growing Type Seven physician brings robust spontaneity and excitement to the workplace and beyond while having a sense of stillness about them.

https://www.thescopeofpractice.com/how-physicians-can-use-the-enneagram-as-a-tool-for-personal-and-professional-development/

#### Slide 27

You may find that Type Sevens:

- are multi-taskers
- are prolific brainstormers
- see connections between ideas
- connections may take them on tangents
- can synthesize disparate ideas together
- enjoy variety
- become distracted when things slow down
- dislike routine, predictability,
- keep plans open-ended

https://compassionatelearning.org/enneagram-personalities-students-copingstrategies-emotionalintelligence-compassion/enneagram-typeseven-student/

#### Slide 28

Type Sevens are a glass-half-full kind of person and bring energy, high spirits, and a sense of adventure and fun to those around them. They will do best in a work environment with a lot of variety, stimulation, and interaction with others. They should be careful not to overload themselves with so many activities that they get scattered, impatient, and drained.

#### Slide 29

Type Eights are the Protective Challengers.

Type Eight physicians are concerned with demonstrating their power. They are the physicians who are known to flaunt their position to the point of being abrasive and bossy.

You may hear a Type Eight saying:

- “Just do what I say.”
- “Do you have a problem with me? Tough luck.”
- “Oh yeah? Come say that to my face.”

They can be combative, abrasive, and quick to remind others about their authority. On the other end of the spectrum, a healthy and growing Type Eight physician can show their power and authority by being vulnerable, and others naturally follow their lead as they move forward.

https://www.thescopeofpractice.com/how-physicians-can-use-the-enneagram-as-a-tool-for-personal-and-professional-development/

#### Slide 30

You may find that Type Eights:

- think rules are seen a limit to independence
- will challenge unfair rules
- feel that unenforced rules “don’t exist”
- push boundaries
- intensity makes Eights feel alive and real
- work with energy while work is challenging
- when bored, tend to look for trouble
- uncomfortable accepting new ideas passively
- need to challenge what they learn, seek proof to back it up

https://compassionatelearning.org/enneagram-personalities-students-copingstrategies-emotionalintelligence-compassion/enneagram-typeeight-student/

#### Slide 31

Type Eights are no strangers to hard work, and may put in longer hours than colleagues. However, know that they’re human too, and need rest and healthy limits. If you’re an eight, take a look at your weekly schedule. Are you overdoing it? Is there any downtime?

If you tend to be overly self-sufficient, see where you might allow others to meet some of your needs and provide support for you.

https://doctorscrossing.com/burning-out-recommendations-for-your-personality-type/

#### Slide 32

Type Nines are the Accommodating Companion

The Enneagram Type Nine physician is concerned with having peace and connection. They are the physicians who mediate in arguments between their fellow colleagues at work or ignore blatant problems in their practice or hospital by sweeping them under the rug.

You may hear a Type Nine say:

- “This is fine.”
- “I try not to think about that.”
- “Let’s not talk about that.”

They may deny real problems by escaping into daydream scenarios. Healthy and growing Type Nines express harmony and peace even in the face of problems and conflicts and are a harbinger of resolutions.

https://www.thescopeofpractice.com/how-physicians-can-use-the-enneagram-as-a-tool-for-personal-and-professional-development/

#### Slide 33

Type Nines like working harmoniously in groups. You may find that Type Nines:

- like all parts fit together in a harmonious way
- sometimes hard to know which pieces are important or not
- hard to prioritize work, it will get done when it gets done
- low stress, little conflict
- high energy environment can be draining
- may take frequent breaks, tune out
- prefer working in groups
- feel the wholeness, unity of the group

https://compassionatelearning.org/enneagram-personalities-students-copingstrategies-emotionalintelligence-compassion/enneagram-typenine-student/

#### Slide 34

Type Nines bring a calm, accepting energy to the workplace and like to be in a comfortable environment where they feel connected to others and valued. They listen deeply and have a gift for seeing things from someone else’s perspective without judgment.

Inertia can take over and days can turn into years. Procrastination is rarely due to laziness. There is usually some underlying fear, concern, or false belief that is maintaining the status quo

https://doctorscrossing.com/burning-out-recommendations-for-your-personality-type/

#### Slide 35

Now that we have some basic understanding of each of the enneagram types, we will now divide into small groups based on type for further discussion.

### Appendix 3 Enneagram Subtypes

[Table t2-jetem-8-4-l1] [Table t3-jetem-8-4-l1] [Table t4-jetem-8-4-l1] [Table t5-jetem-8-4-l1] [Table t6-jetem-8-4-l1]

**Table t2-jetem-8-4-l1:**

One – The Reformer		Two – The Helper	
*Description*	Ones desire rules and doing things correctly. They are therefore strict with themselves and hold others to an equally high standard. They fear being imperfect.	*Description*	Twos want to be liked and helpful to others. This helps with their sense of belonging. Twos fear being unlovable.
*Basic Fear*	Being bad, imbalanced, defective, corrupt	*Basic Fear*	Being unloved
*Basic Desire*	Have integrity, to be good	*Basic Desire*	Feel love
*In search of*	Integrity and improvement	*In search of*	Intimacy
*Healthy Sense of Self*	I am a reasonable, objective person	*Healthy Sense of Self*	I am a caring, loving person
*Hidden Complaint*	Others should listen to me because I’m right most of the time	*Hidden Complaint*	Others take me for granted because I’m always loving
*Virtue*	Serenity	*Virtue*	Humility
*Fixation*	Resentment	*Fixation*	Flattery
*Deadly Sin*	Anger	*Deadly Sin*	Pride
*Main Temptation*	Extreme sense of personal moral obligation	*Main Temptation*	Without needs, good intentions
*Saving Grace*	Objective and sensible	*Saving Grace*	Empathy

**Table t3-jetem-8-4-l1:**

Three – The Achiever		Four – The Individualist	
*Description*	Threes desire to be successful and admired by others. They are very conscious of their image. Threes fear failure and not being valuable.	*Description*	Fours want to be unique and experience emotions. Fours fear they are outsiders.
*Basic Fear*	Being worthless	*Basic Fear*	Being unidentifiable or insignificant
*Basic Desire*	Feel valuable	*Basic Desire*	Be themselves
*In search of*	Acceptance and validation of actions	*In search of*	Identity
*Healthy Sense of Self*	I am an outstanding, effective person	*Healthy Sense of Self*	I am an intuitive, sensitive person
*Hidden Complaint*	Others are jealous of me because I’m a superior person	*Hidden Complaint*	I don’t fit in because others are different from me
*Virtue*	Truth and Authenticity	*Virtue*	Levelheadedness
*Fixation*	Vanity	*Fixation*	Melancholy
*Deadly Sin*	Deceit	*Deadly Sin*	Envy
*Main Temptation*	Push themselves to be “the best”	*Main Temptation*	To overuse imagination in search of self
*Saving Grace*	Desire to be accepted	*Saving Grace*	Self-Awareness

**Table t4-jetem-8-4-l1:**

Five – The Investigator		Six – The Loyalist	
*Description*	Fives seek knowledge. They are more comfortable with data. Their biggest fear is being overwhelmed by needs of themselves or others.	*Description*	Sixes desire security and safety. They want to be prepared for all situations. Their fear is to be unprepared.
*Basic Fear*	Helpless, incompetent	*Basic Fear*	Being without Support
*Basic Desire*	Capable and competent	*Basic Desire*	Guidance and support
*In search of*	Knowledge mastery	*In search of*	Safety and security
*Healthy Sense of Self*	I am a highly knowledgeable person	*Healthy Sense of Self*	I am loyal, dependable person
*Hidden Complaint*	I am so intelligent others can’t understand me.	*Hidden Complaint*	I do what I’m supposed to do but others don’t
*Virtue*	Nonattachment	*Virtue*	Courage
*Fixation*	Stinginess	*Fixation*	Worrying
*Deadly Sin*	Greed	*Deadly Sin*	Anxiety
*Main Temptation*	Replacing experiencing life with concepts	*Main Temptation*	Seek others for reassurance
*Saving Grace*	Awareness of own distortions	*Saving Grace*	Relationships

**Table t5-jetem-8-4-l1:**

Seven – The Enthusiast		Eight– The Challenger	
*Description*	Sevens desire fun and adventure. They are bored easily. They seek to avoid emotional pain by staying busy and avoiding negative situations.	*Description*	Eights are strong and powerful. They stand up for what they believe in. They work to control their environment to avoid their greatest fear which is to be powerless.
*Basic Fear*	Being trapped in boredom	*Basic Fear*	Being controlled or violated
*Basic Desire*	Feel satisfied and content	*Basic Desire*	Protect themselves
*In search of*	Fulfillment and adventure	*In search of*	Survival
*Healthy Sense of Self*	I am a happy, enthusiastic person	*Healthy Sense of Self*	I am a strong, assertive person
*Hidden Complaint*	I’d be happier if others give me what I want	*Hidden Complaint*	Others take advantage of me
*Virtue*	Clearheadedness	*Virtue*	Innocence
*Fixation*	Anticipation	*Fixation*	Venegance
*Deadly Sin*	Gluttony	*Deadly Sin*	Forcefulness
*Main Temptation*	To look for fulfillment elswhere	*Main Temptation*	To be self-sufficient and not rely on others
*Saving Grace*	Knowing what is next	*Saving Grace*	Knowing their forcefulness threatens their survival

**Table t6-jetem-8-4-l1:**

Nine– The Peacemaker	
*Description*	Nines are flexible and like for others around them to set the agenda. They fear prioritizing their own needs. They may be called passive.
*Basic Fear*	Loss, separation, conflict
*Basic Desire*	Peace of mind
*In search of*	Harmony and stability
*Healthy Sense of Self*	I am an easygoing, peaceful person.
*Hidden Complaint*	Others pressure me to change.
*Virtue*	Action
*Fixation*	Daydreaming
*Deadly Sin*	Disengagement
*Main Temptation*	Avoiding conflicts
*Saving Grace*	Value relationships

### Appendix 4: Discussion Questions for Small Groups

Focusing on best communication methods, meeting and email structure, feedback, burn-out risks, work environment, and interpersonal interactions.

1. What characteristics of your enneagram type stood out to you?
2. What parts of the characteristics surprised you?
3. What frustrates you when working in a team? When do you feel most productive in a team? How does this apply in the Emergency Department?
4. How do you prefer for people to communicate with you about work?
5. Think about communication from the departmental leadership team. What information do you desire in emails? What is your ideal meeting structure?
6. How would you like to receive feedback or constructive criticism?
7. What part of your personality do you feel puts you at risk for frustration at work and burnout?
8. How will you use your enneagram personality traits as a guide for your future work environment?

### Appendix 5 Enneagram in EM Session Evaluation

1. I now self-identify with a primary enneagram type and can see parts of my personality and behavior represented in this type.
  Disagree – Somewhat disagree – Somewhat agree – Agree
2. I can name the basic fears, desires, and motivations of my enneagram type.
  Disagree – Somewhat disagree – Somewhat agree – Agree
3. I now can identify and describe possible struggles in interacting with other disparate enneagram types.
  Disagree – Somewhat disagree – Somewhat agree – Agree
4. I am aware of strategies for succeeding in facing conflict and interacting with other team members.
  Disagree – Somewhat disagree – Somewhat agree – Agree
5. The information today was presented in an engaging and informative way.
  Disagree – Somewhat disagree – Somewhat agree – Agree
