# Supplementary material for: Enneagram in EM
Source: J Educ Teach Emerg Med. 2023 Oct 31;8(4):L1–L22. doi: 10.21980/J8ZM0G (PMC10631809; doi:10.21980/J8ZM0G)
Supplement: Supplementary file 1 [file jetem-8-4-L1-supp1.pptx]

## Slide 1
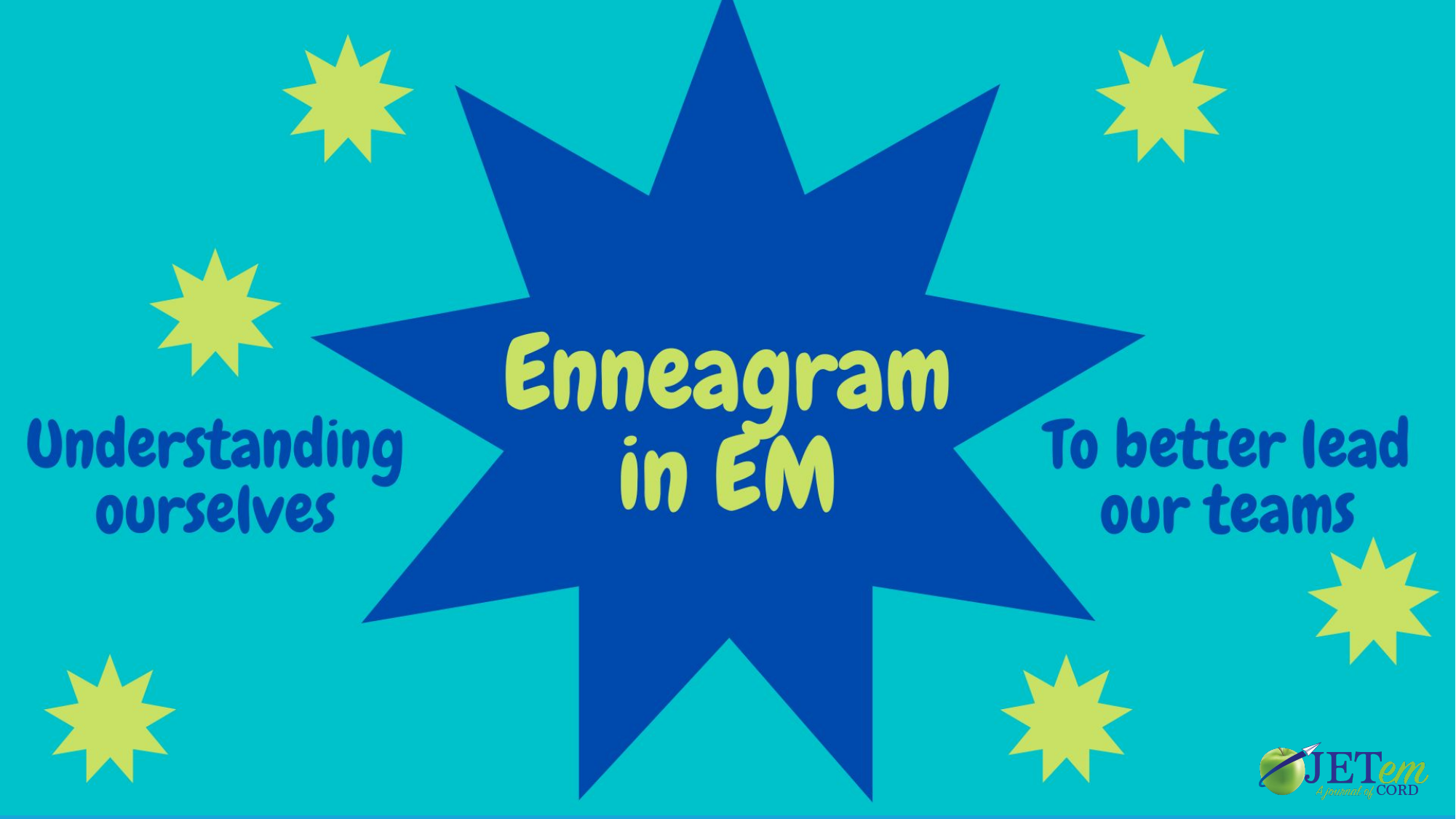

## Slide 2
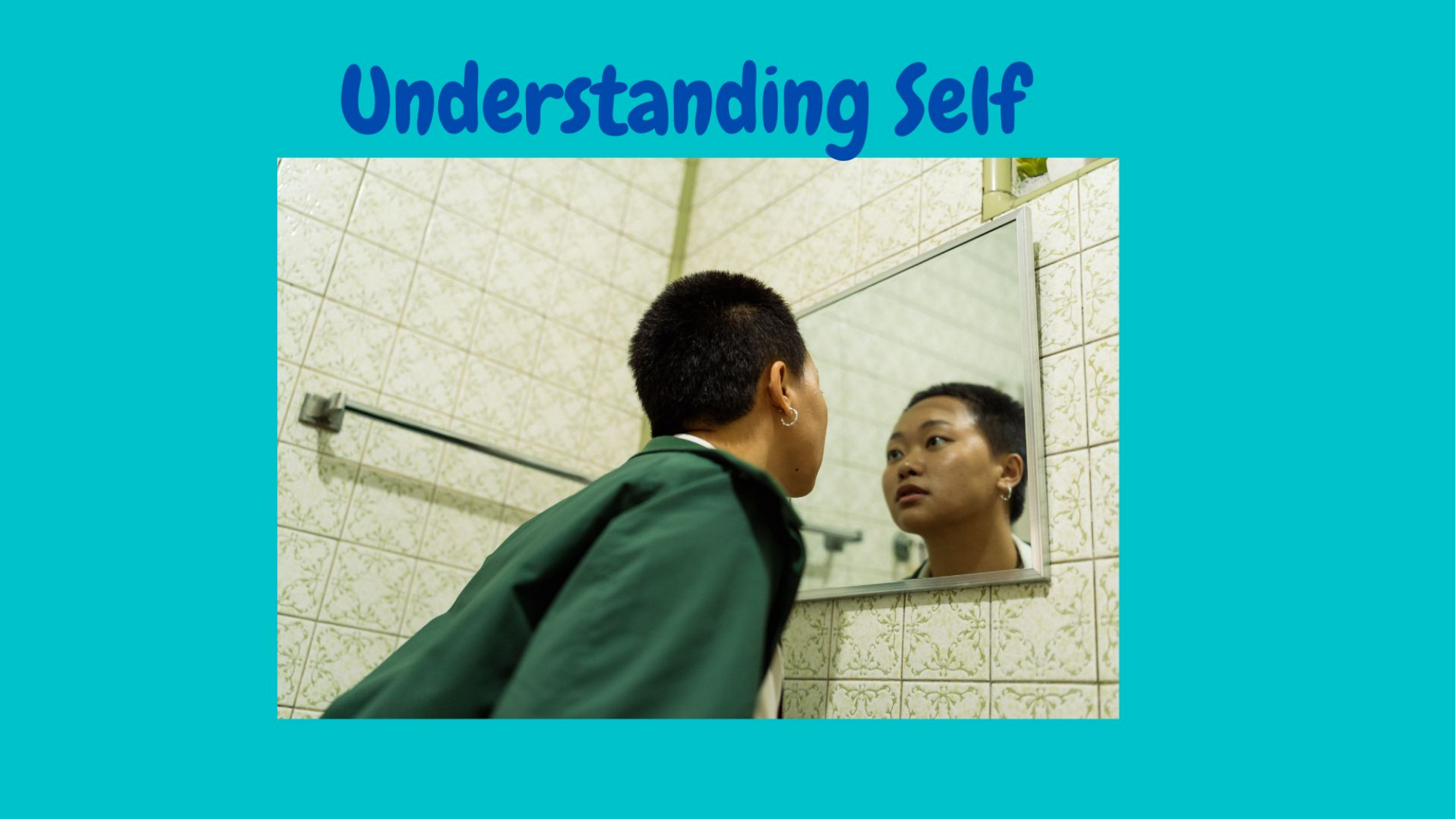

## Slide 3
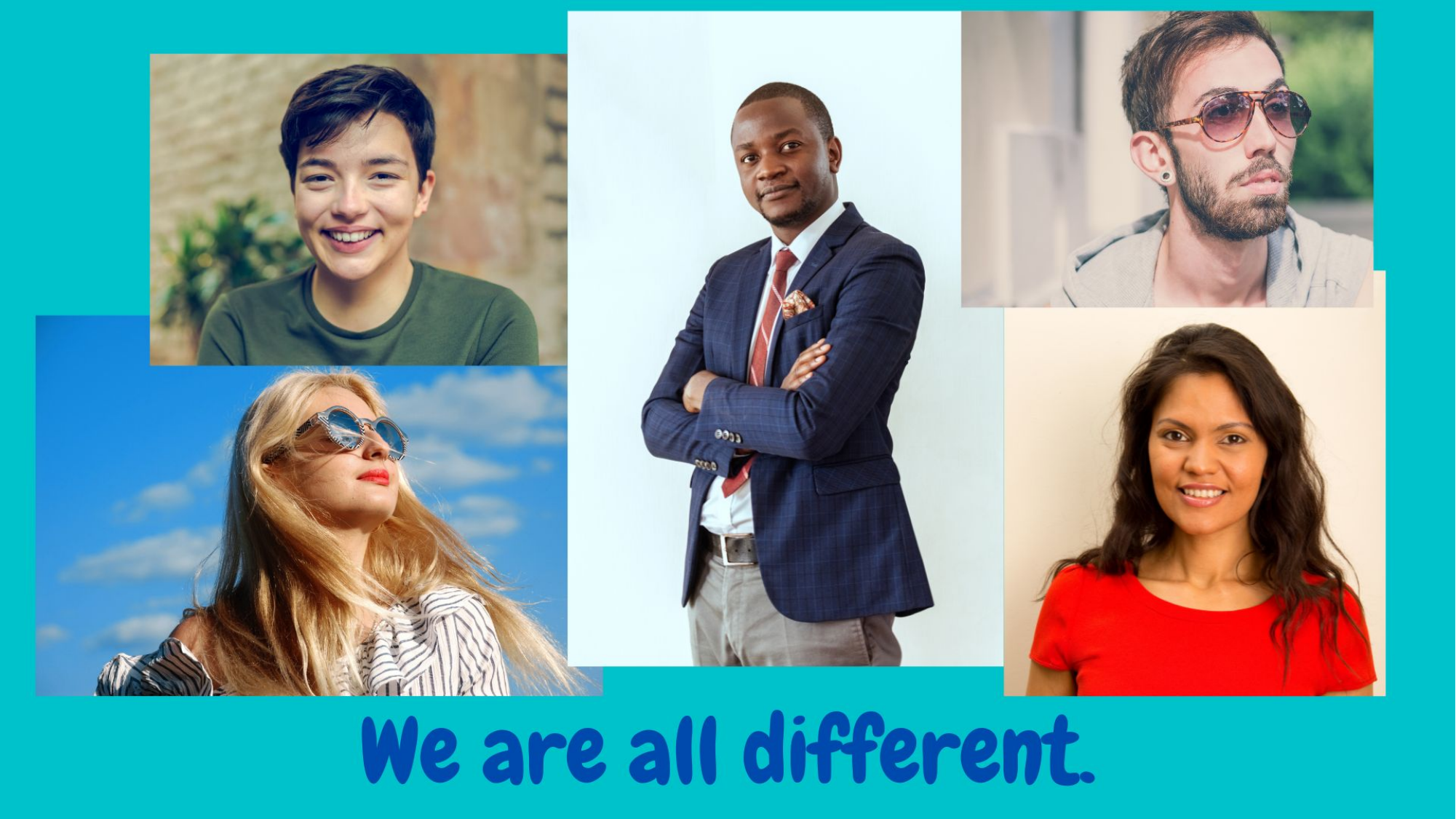

## Slide 4
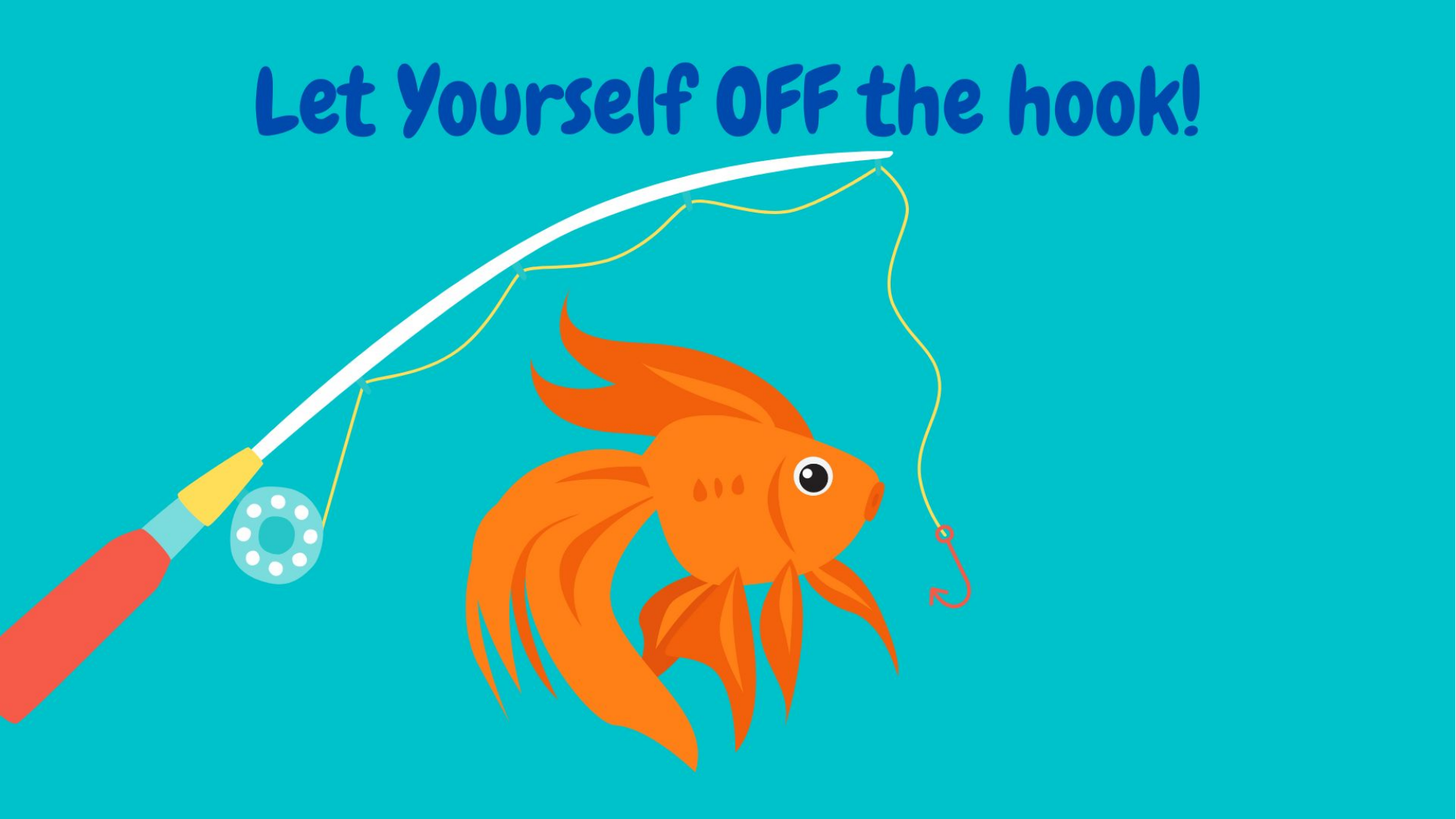

## Slide 5
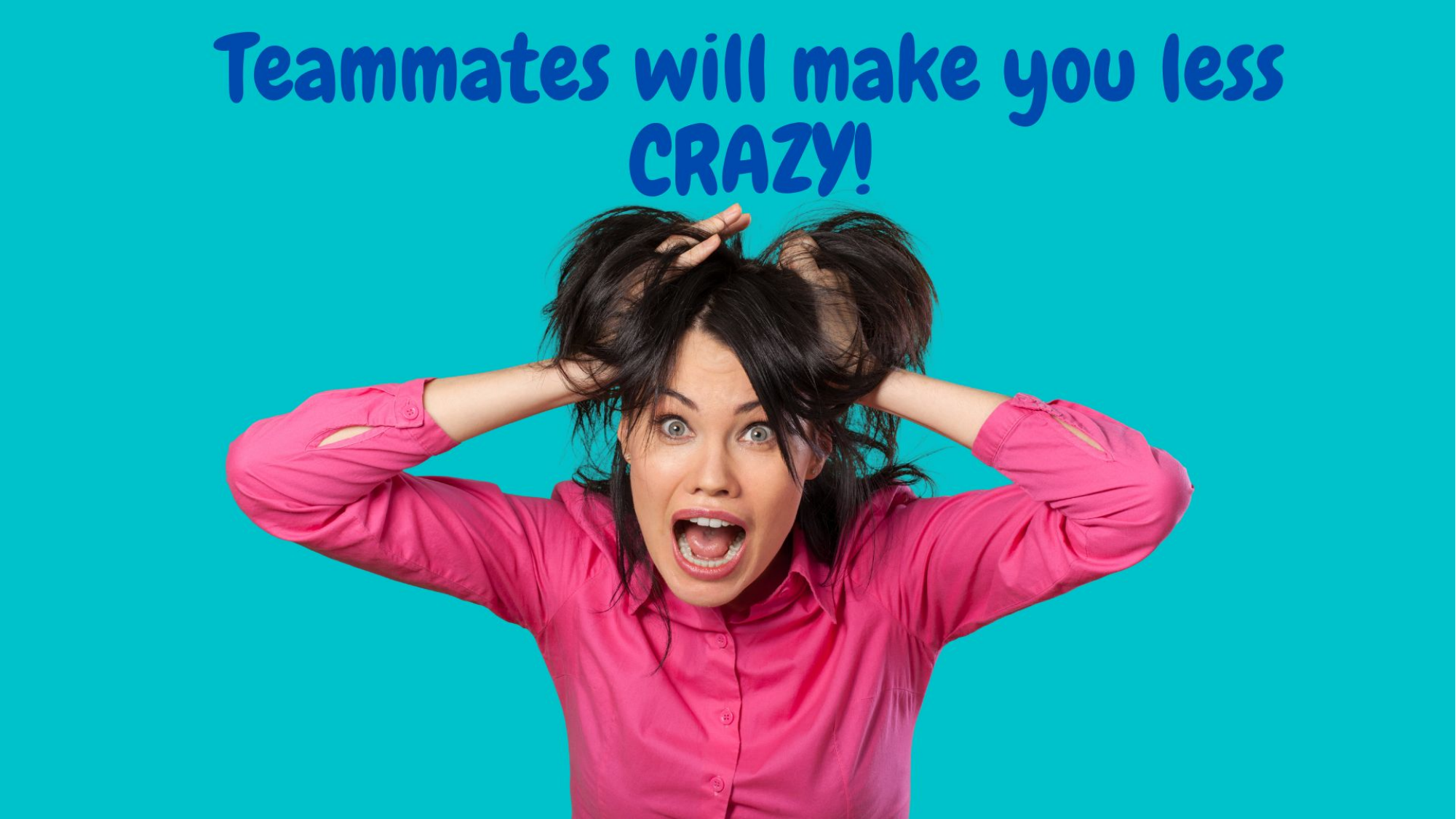

## Slide 6
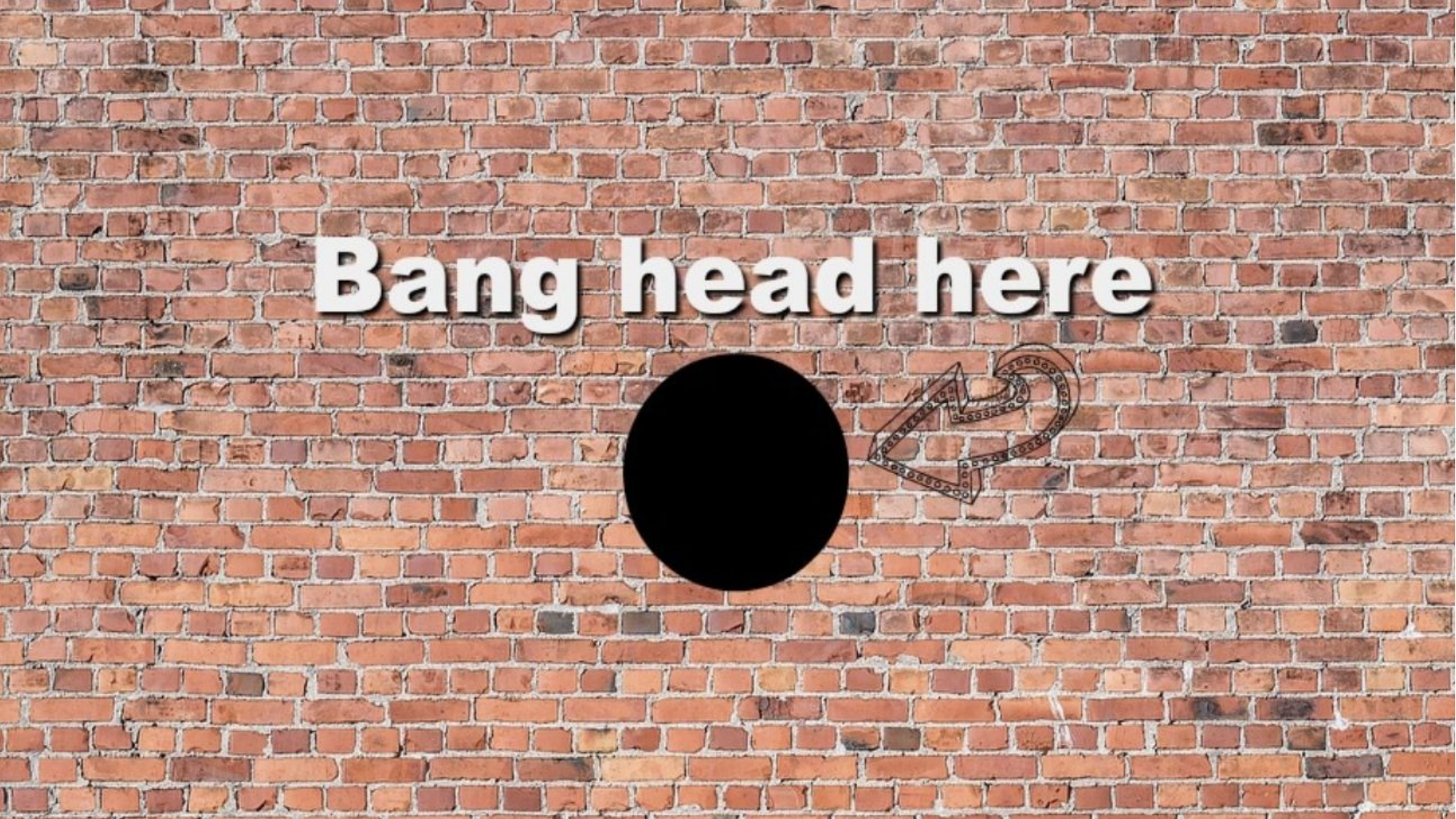

## Slide 7
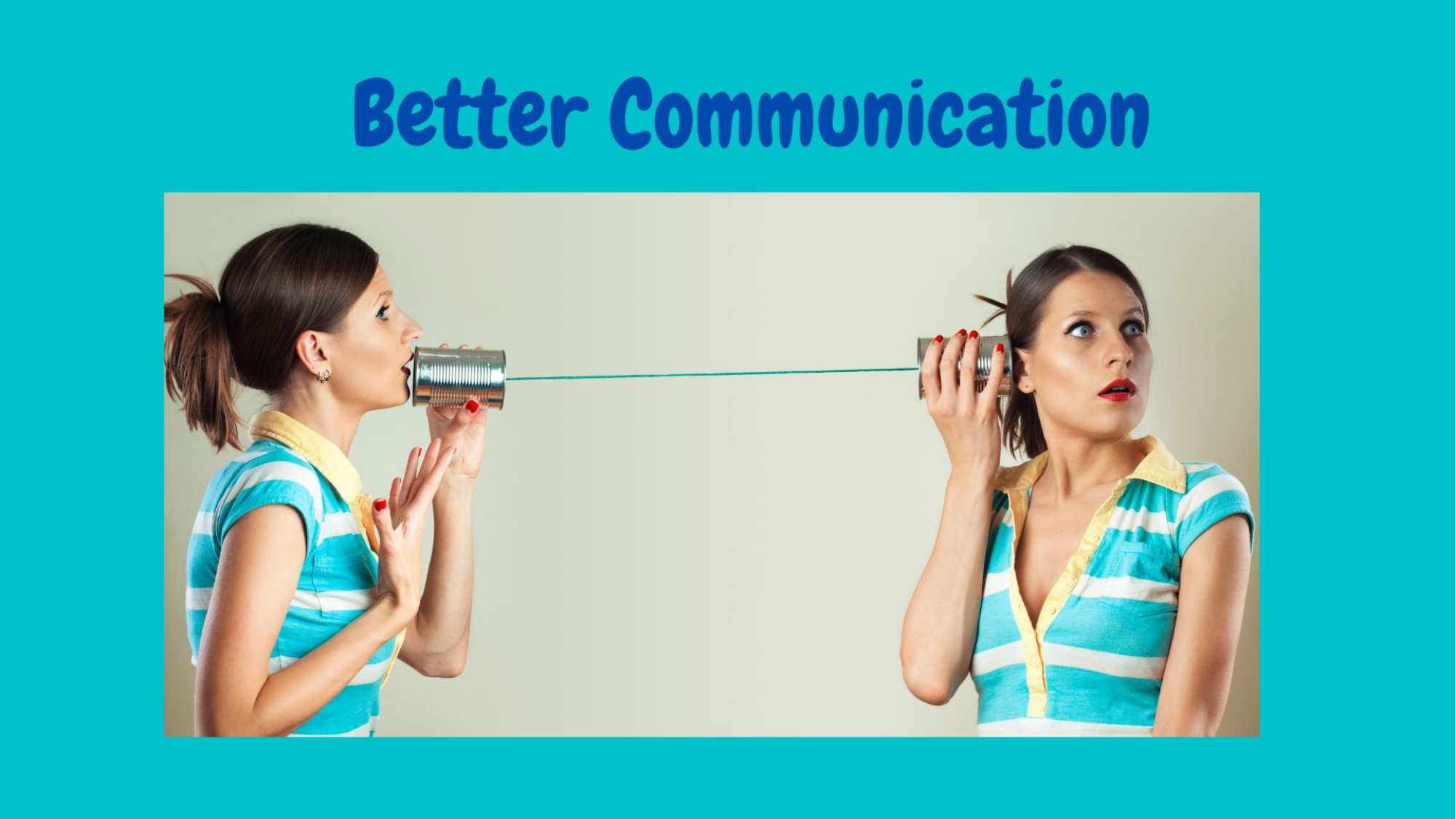

## Slide 8
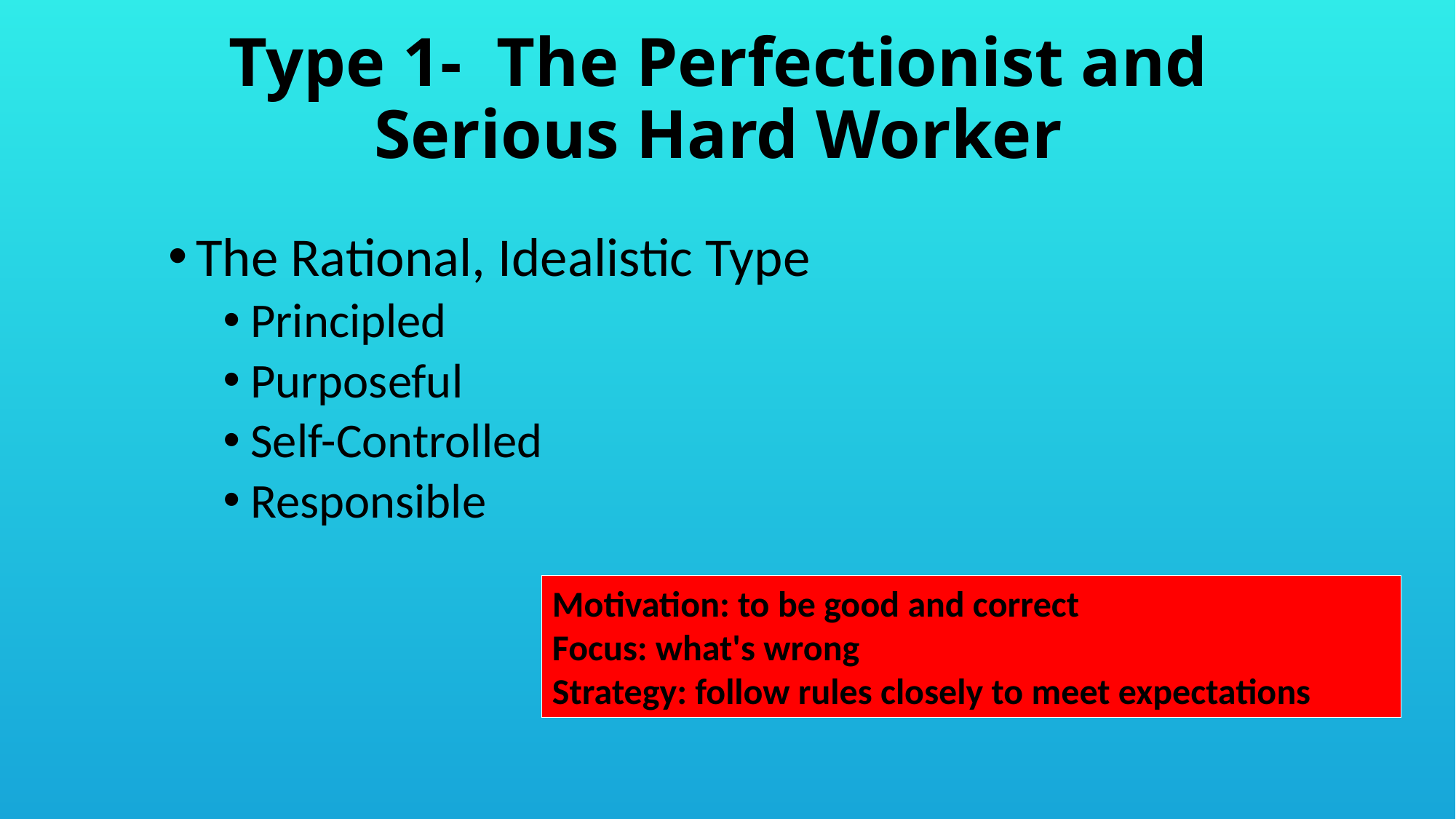

# Type 1- The Perfectionist and Serious Hard Worker
The Rational, Idealistic Type
Principled
Purposeful
Self-Controlled
Responsible
Motivation: to be good and correct
Focus: what's wrong
Strategy: follow rules closely to meet expectations

## Slide 9
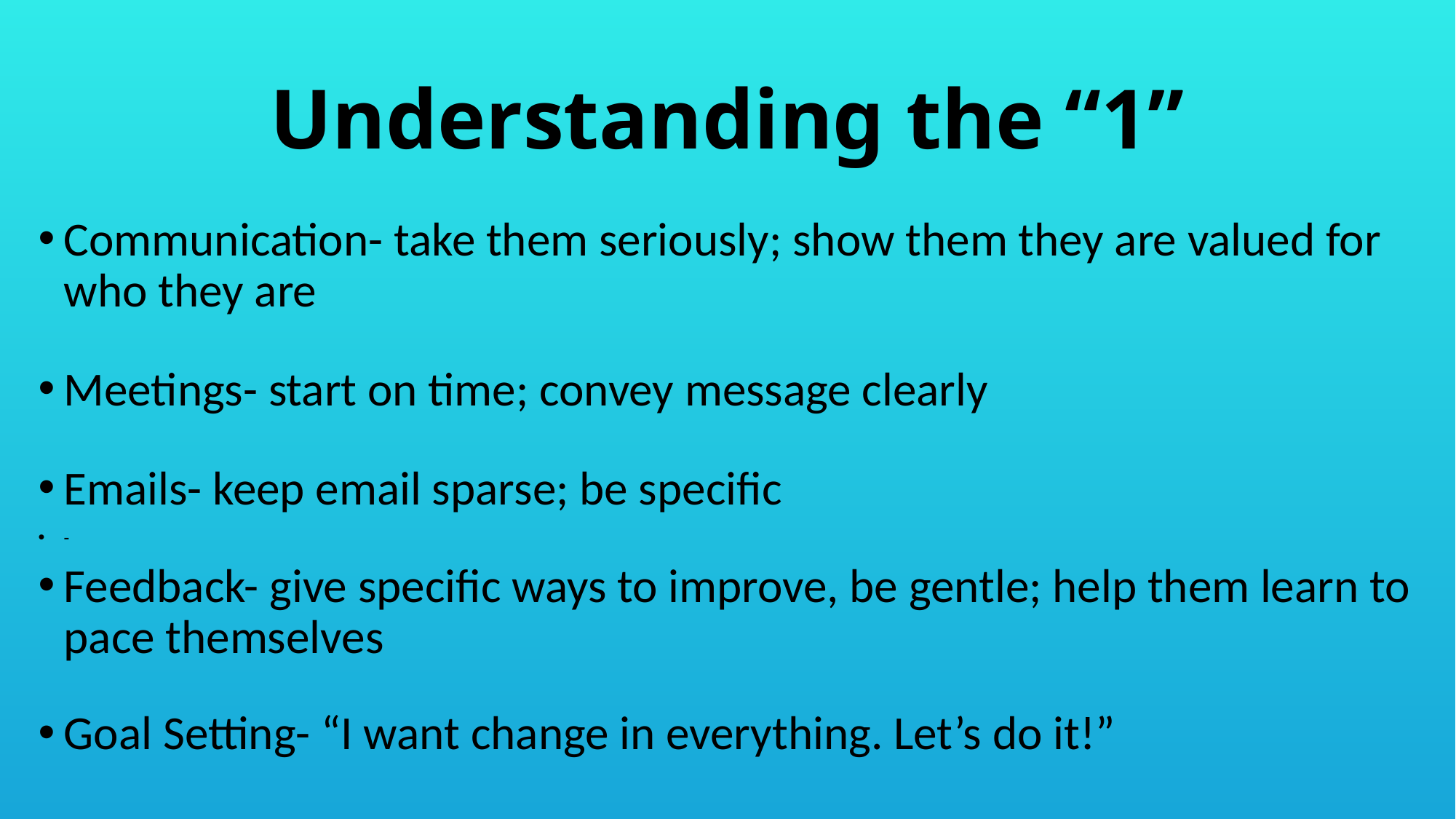

# Understanding the “1”
Communication- take them seriously; show them they are valued for who they are
Meetings- start on time; convey message clearly
Emails- keep email sparse; be specific
-
Feedback- give specific ways to improve, be gentle; help them learn to pace themselves
Goal Setting- “I want change in everything. Let’s do it!”

## Slide 10
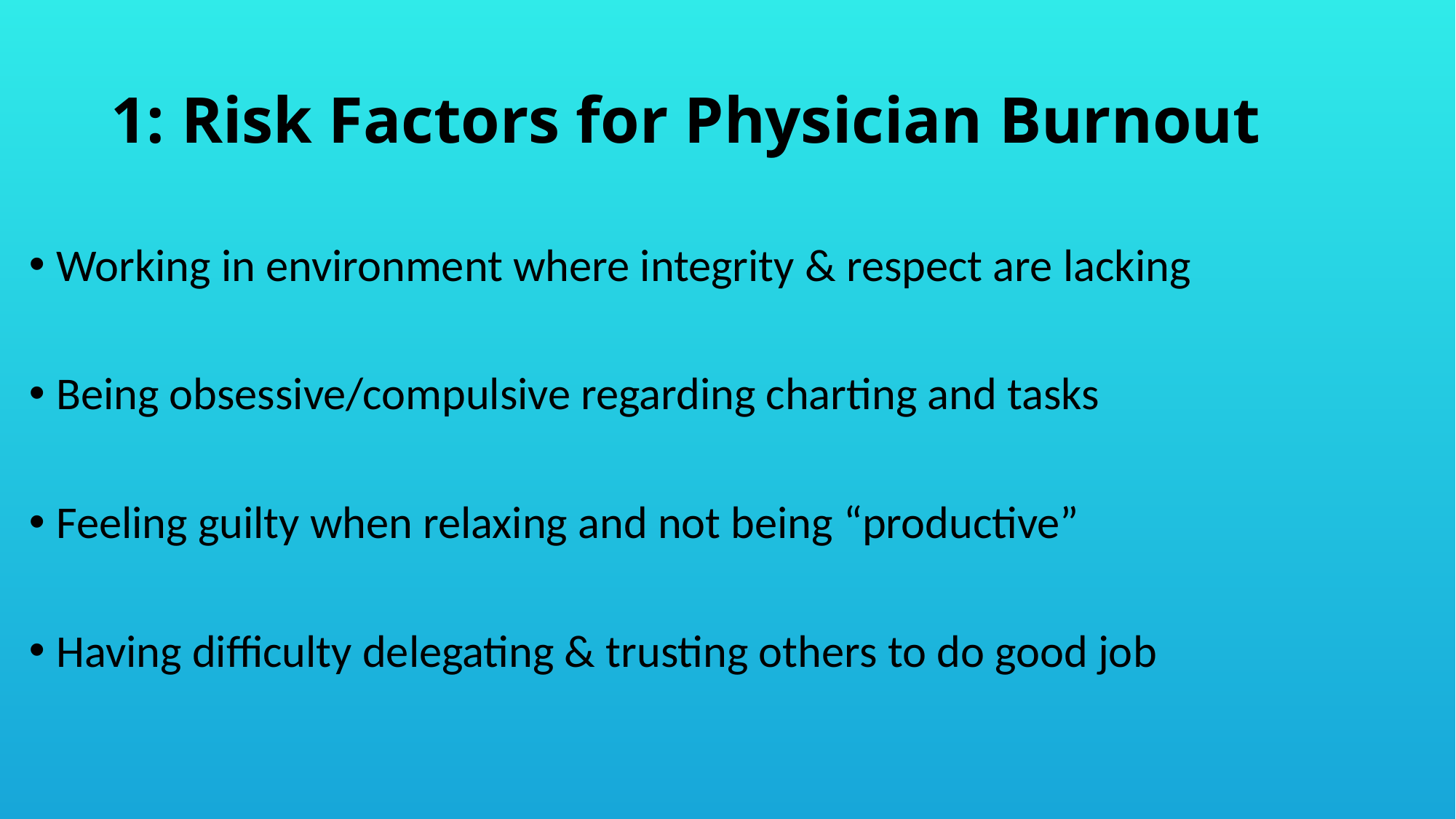

# 1: Risk Factors for Physician Burnout
Working in environment where integrity & respect are lacking
Being obsessive/compulsive regarding charting and tasks
Feeling guilty when relaxing and not being “productive”
Having difficulty delegating & trusting others to do good job

## Slide 11
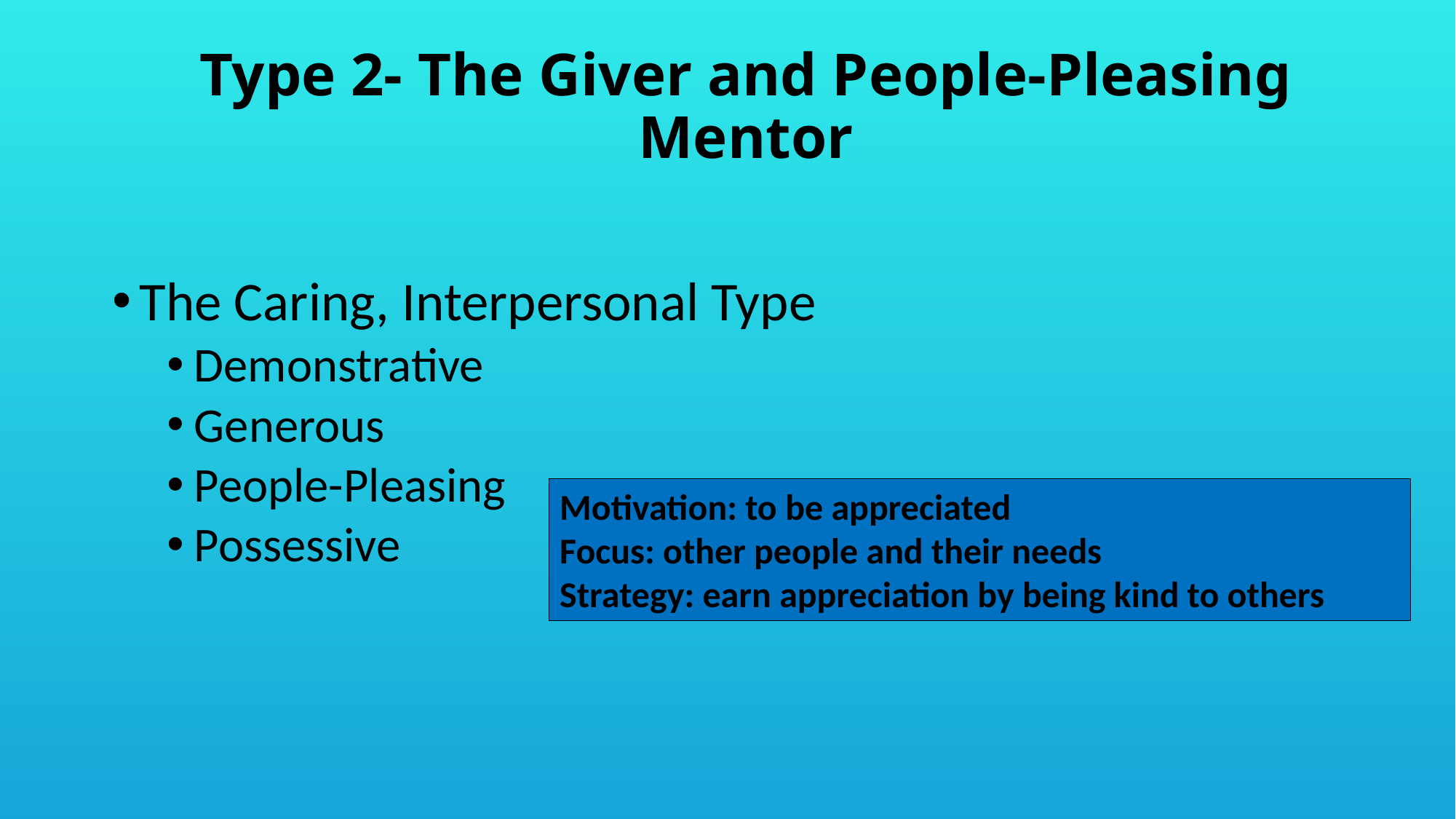

# Type 2- The Giver and People-Pleasing Mentor
The Caring, Interpersonal Type
Demonstrative
Generous
People-Pleasing
Possessive
Motivation: to be appreciated
Focus: other people and their needs
Strategy: earn appreciation by being kind to others

## Slide 12
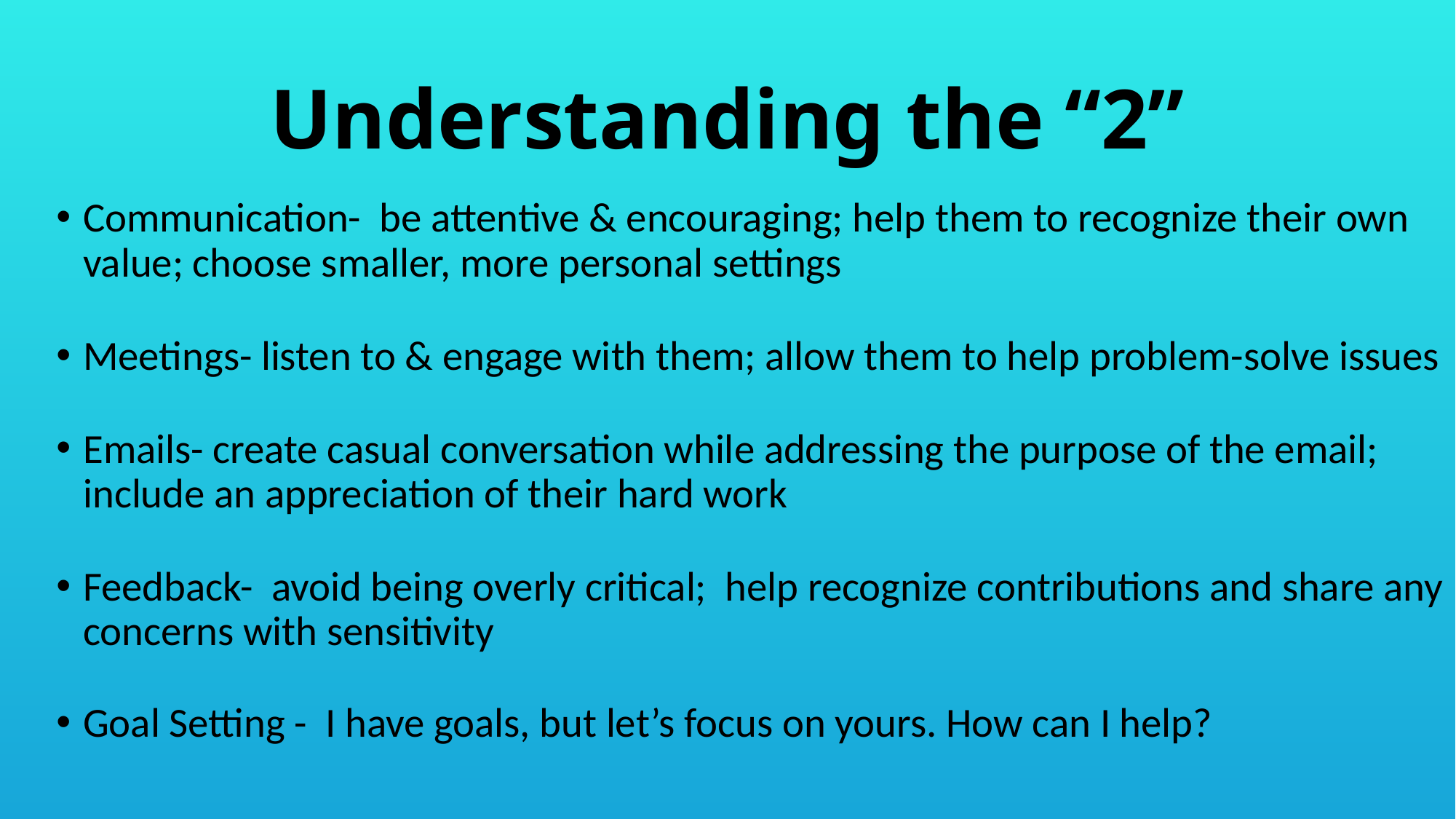

# Understanding the “2”
Communication- be attentive & encouraging; help them to recognize their own value; choose smaller, more personal settings
Meetings- listen to & engage with them; allow them to help problem-solve issues
Emails- create casual conversation while addressing the purpose of the email; include an appreciation of their hard work
Feedback- avoid being overly critical; help recognize contributions and share any concerns with sensitivity
Goal Setting - I have goals, but let’s focus on yours. How can I help?

## Slide 13
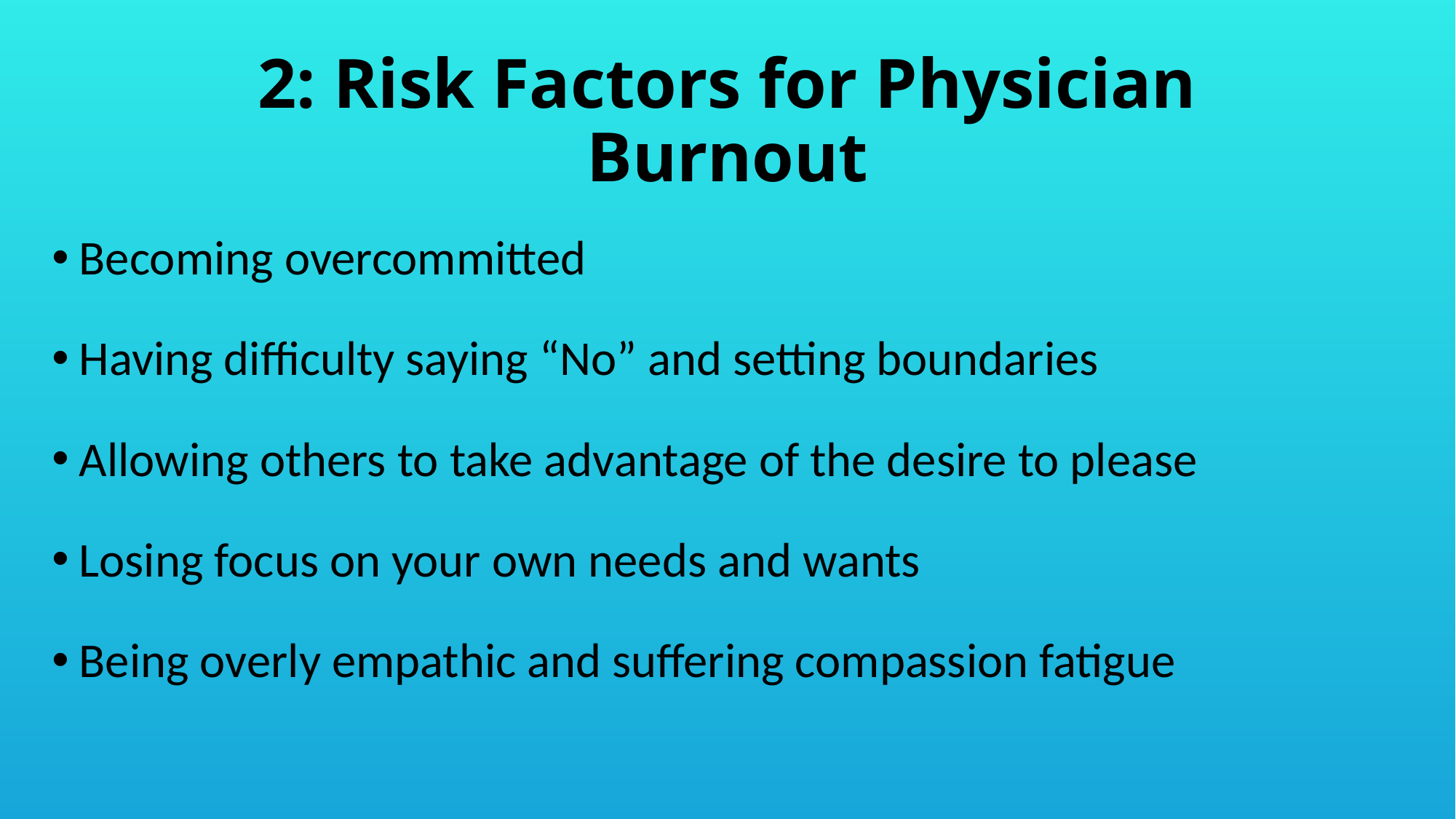

# 2: Risk Factors for Physician Burnout
Becoming overcommitted
Having difficulty saying “No” and setting boundaries
Allowing others to take advantage of the desire to please
Losing focus on your own needs and wants
Being overly empathic and suffering compassion fatigue

## Slide 14
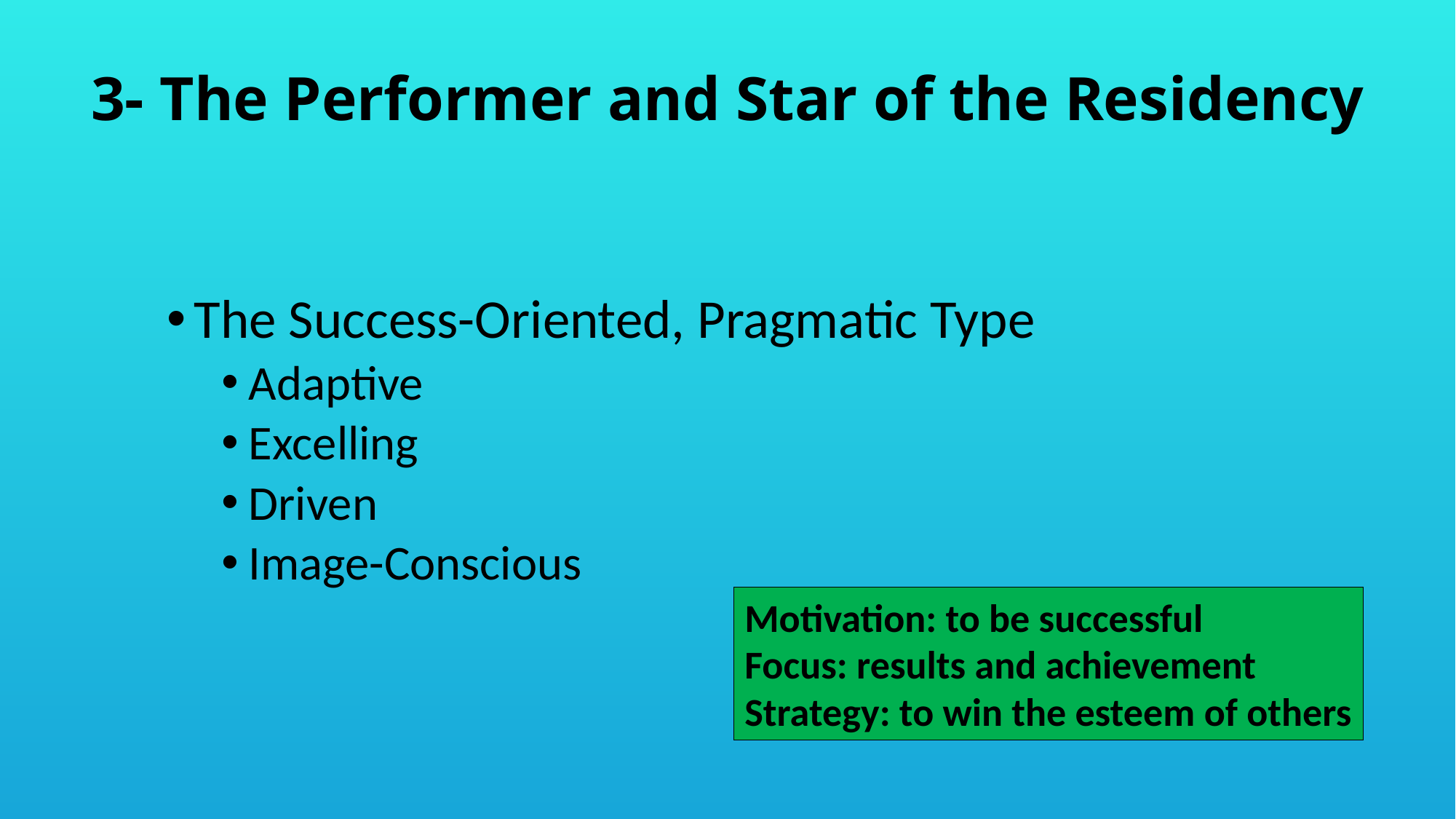

# 3- The Performer and Star of the Residency
The Success-Oriented, Pragmatic Type
Adaptive
Excelling
Driven
Image-Conscious
Motivation: to be successful
Focus: results and achievement
Strategy: to win the esteem of others

## Slide 15
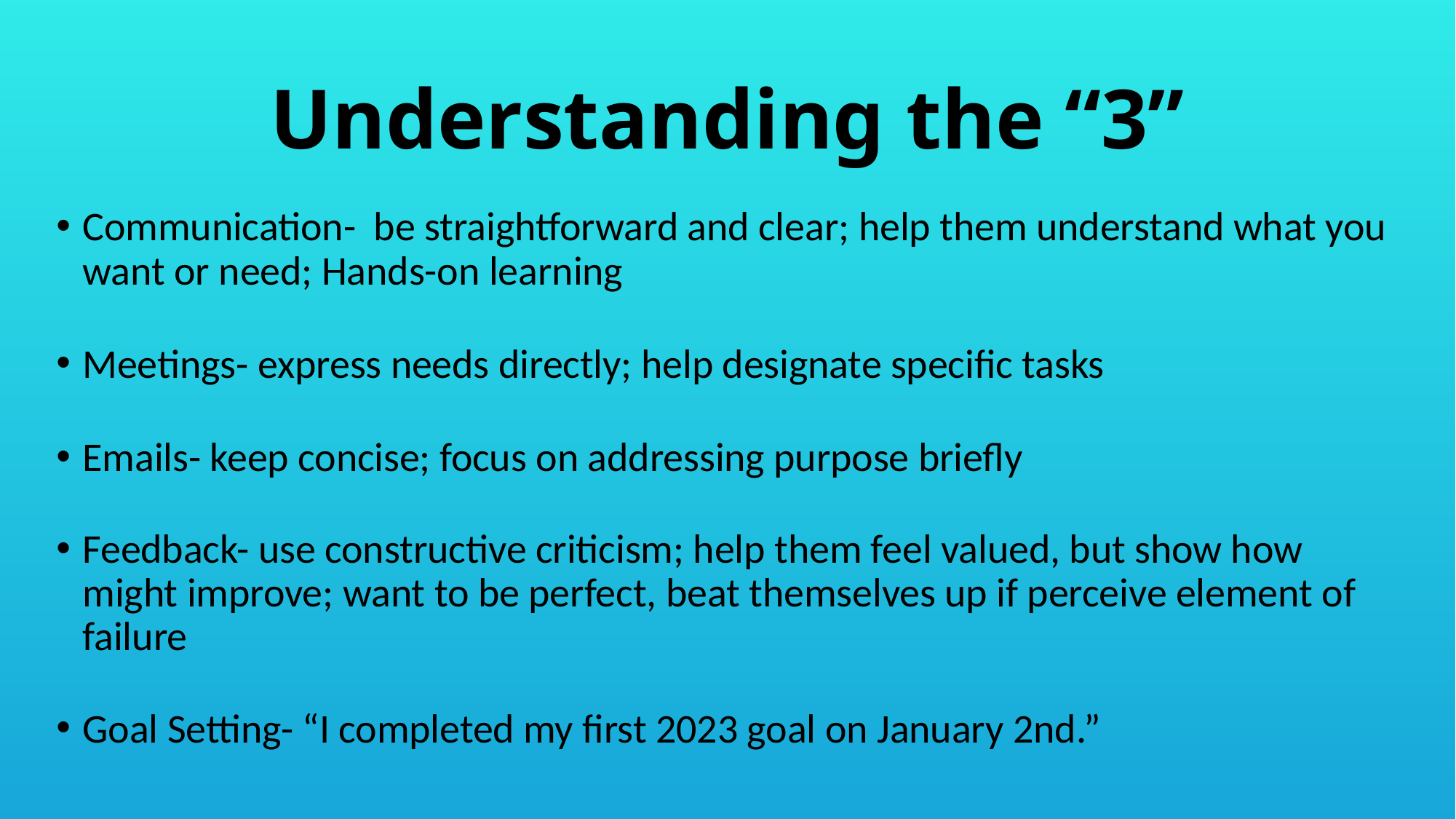

# Understanding the “3”
Communication- be straightforward and clear; help them understand what you want or need; Hands-on learning
Meetings- express needs directly; help designate specific tasks
Emails- keep concise; focus on addressing purpose briefly
Feedback- use constructive criticism; help them feel valued, but show how might improve; want to be perfect, beat themselves up if perceive element of failure
Goal Setting- “I completed my first 2023 goal on January 2nd.”

## Slide 16
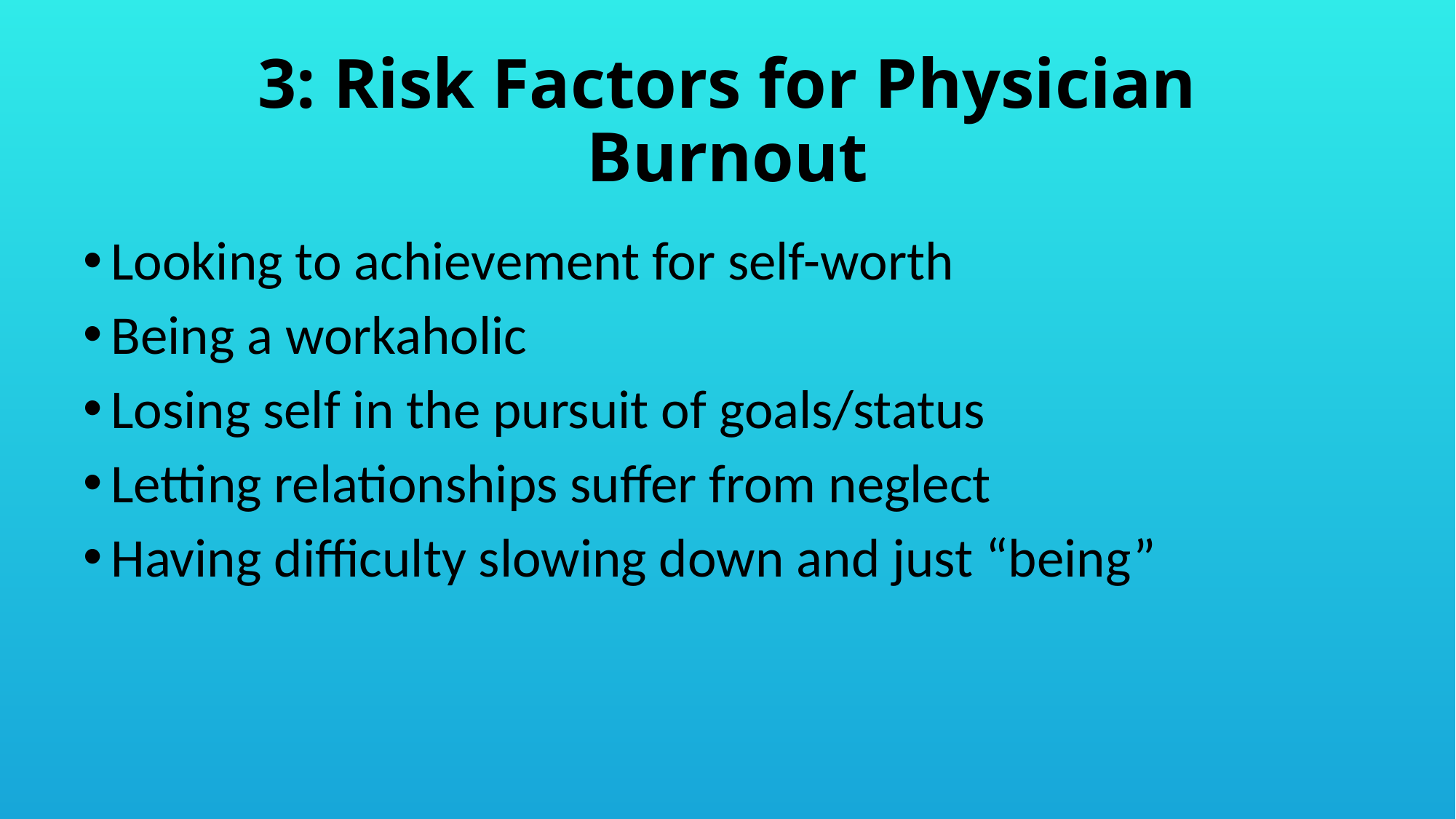

# 3: Risk Factors for Physician Burnout
Looking to achievement for self-worth
Being a workaholic
Losing self in the pursuit of goals/status
Letting relationships suffer from neglect
Having difficulty slowing down and just “being”

## Slide 17
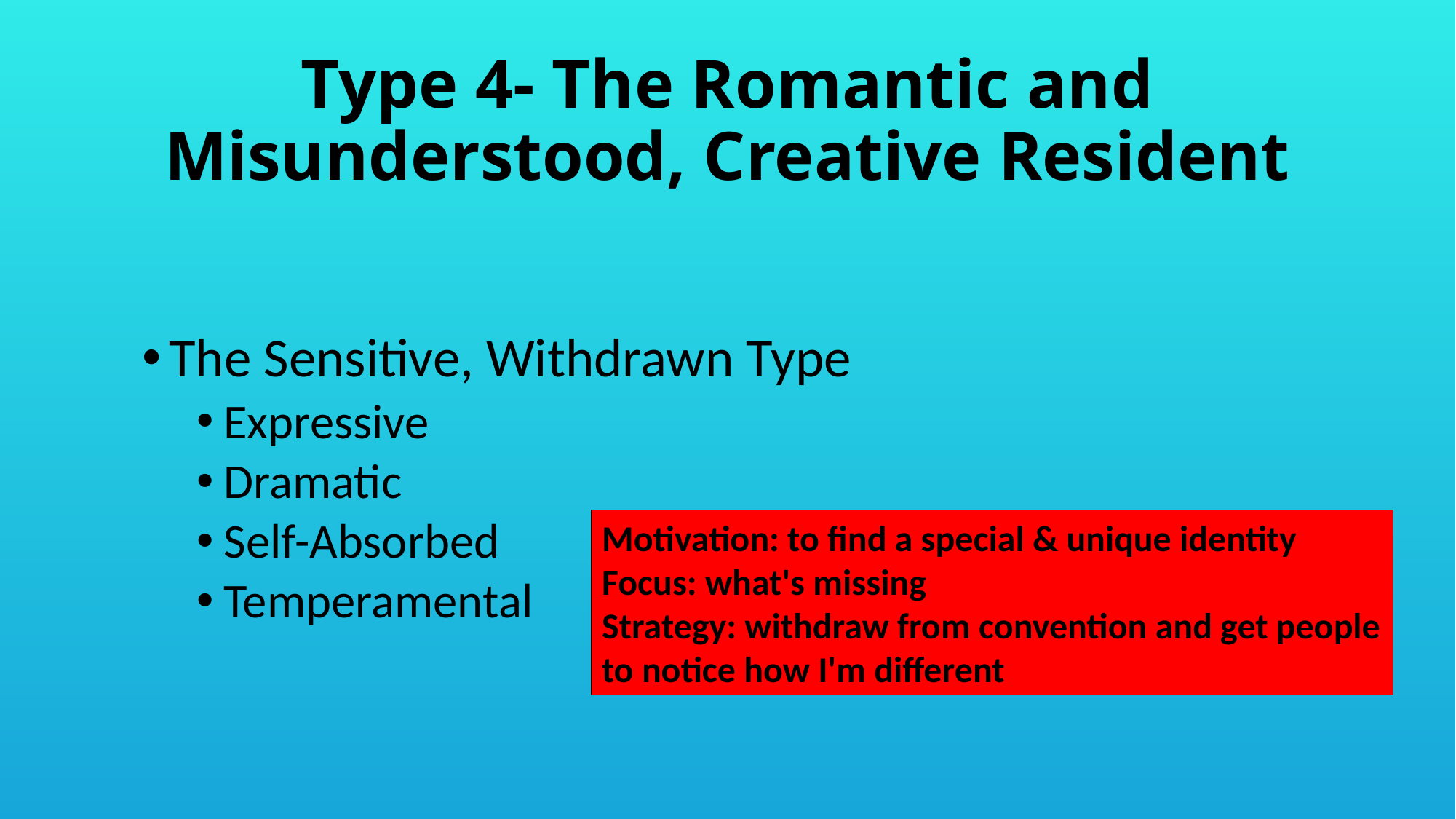

# Type 4- The Romantic and Misunderstood, Creative Resident
The Sensitive, Withdrawn Type
Expressive
Dramatic
Self-Absorbed
Temperamental
Motivation: to find a special & unique identity
Focus: what's missing
Strategy: withdraw from convention and get people to notice how I'm different

## Slide 18
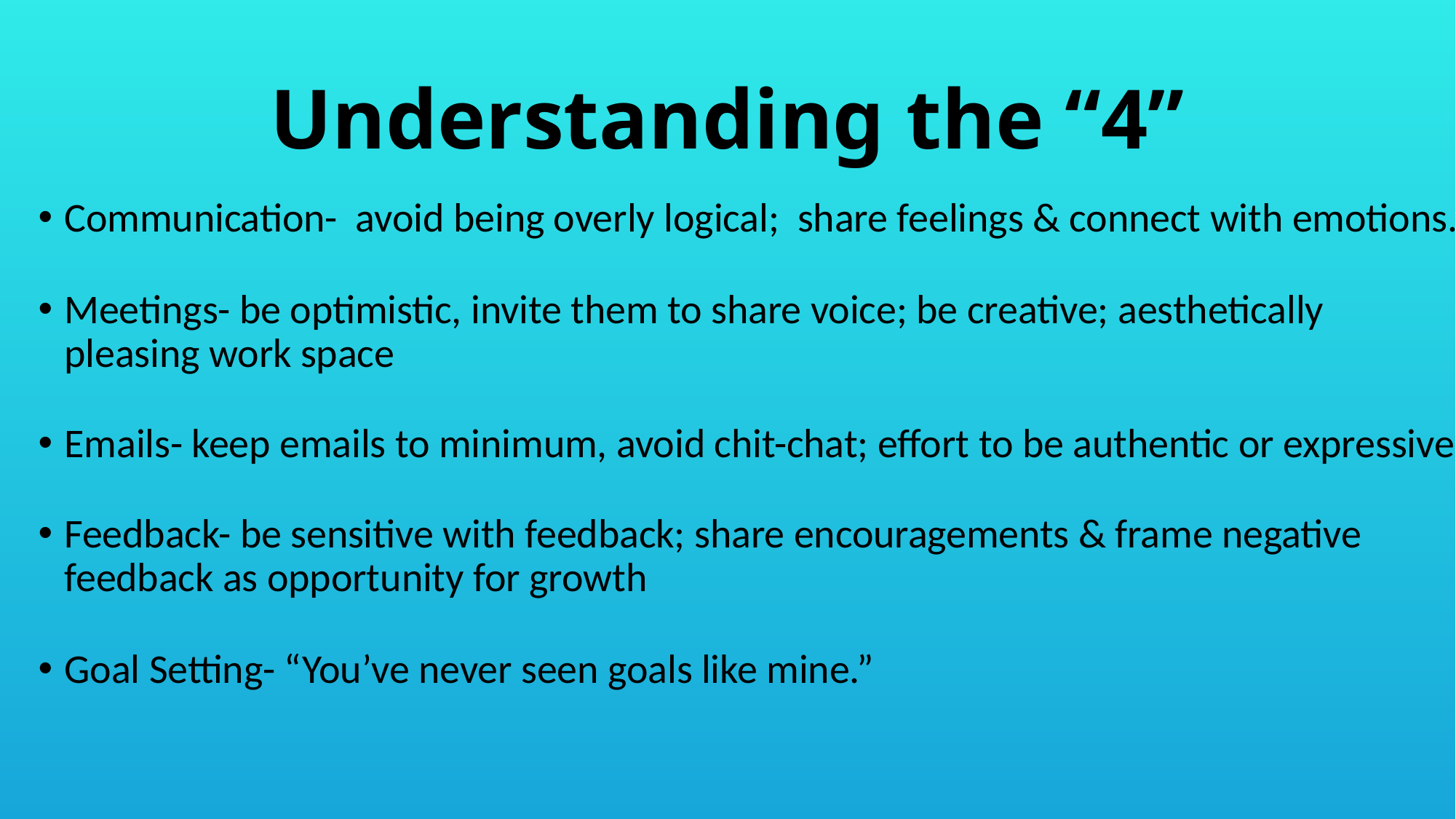

# Understanding the “4”
Communication- avoid being overly logical; share feelings & connect with emotions.
Meetings- be optimistic, invite them to share voice; be creative; aesthetically pleasing work space
Emails- keep emails to minimum, avoid chit-chat; effort to be authentic or expressive
Feedback- be sensitive with feedback; share encouragements & frame negative feedback as opportunity for growth
Goal Setting- “You’ve never seen goals like mine.”

## Slide 19
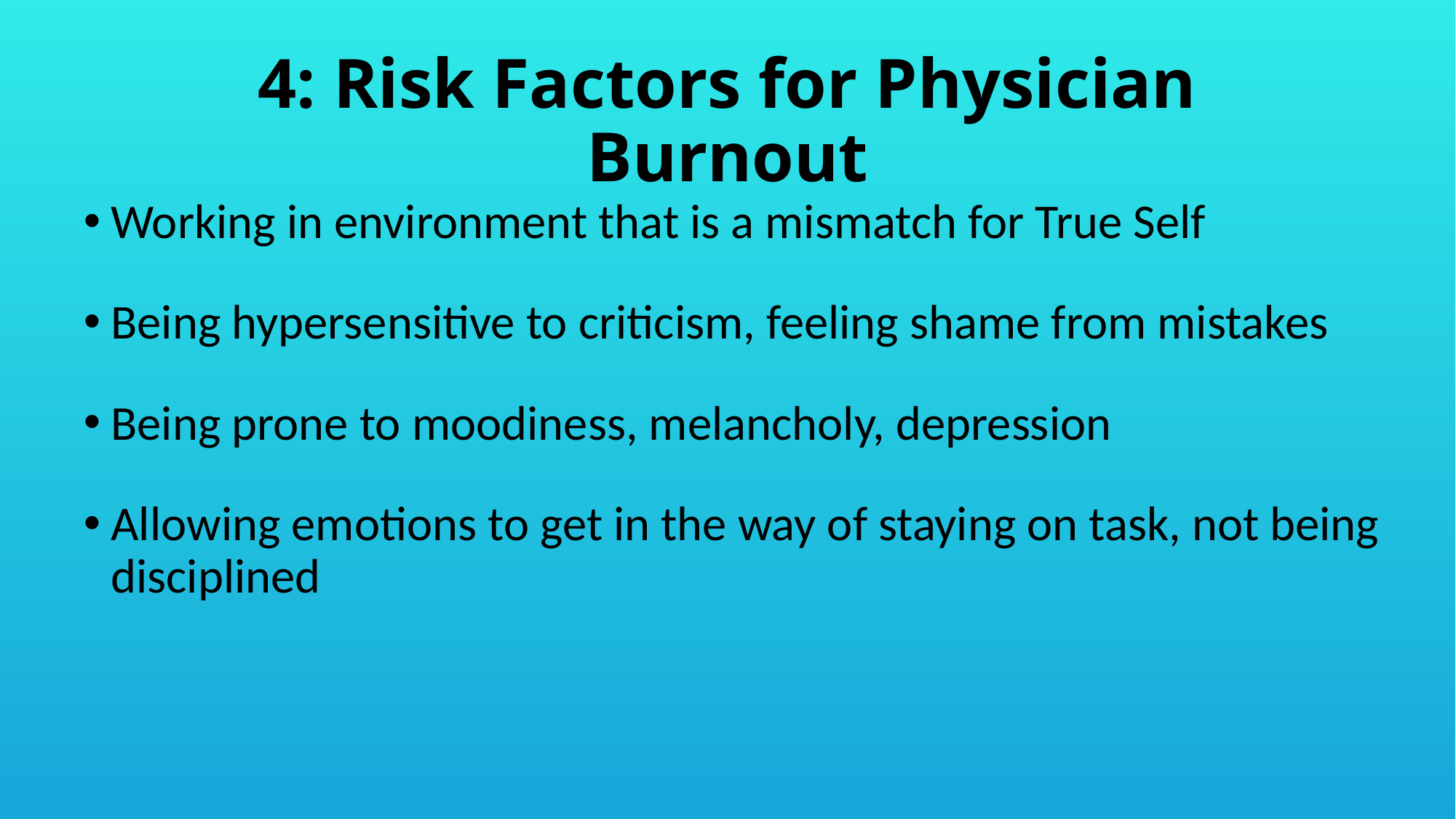

# 4: Risk Factors for Physician Burnout
Working in environment that is a mismatch for True Self
Being hypersensitive to criticism, feeling shame from mistakes
Being prone to moodiness, melancholy, depression
Allowing emotions to get in the way of staying on task, not being disciplined

## Slide 20
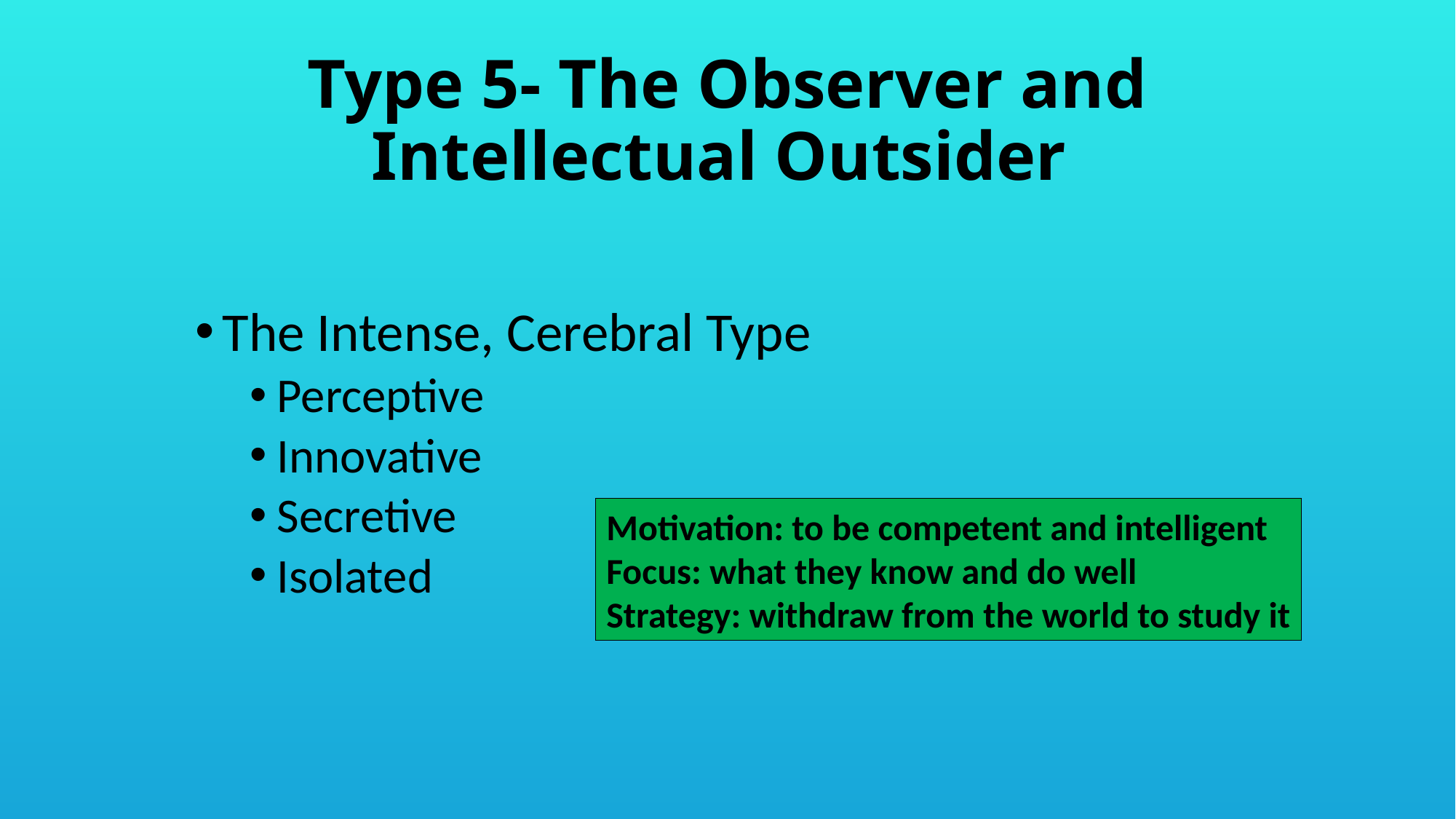

# Type 5- The Observer and Intellectual Outsider
The Intense, Cerebral Type
Perceptive
Innovative
Secretive
Isolated
Motivation: to be competent and intelligent
Focus: what they know and do well
Strategy: withdraw from the world to study it

## Slide 21
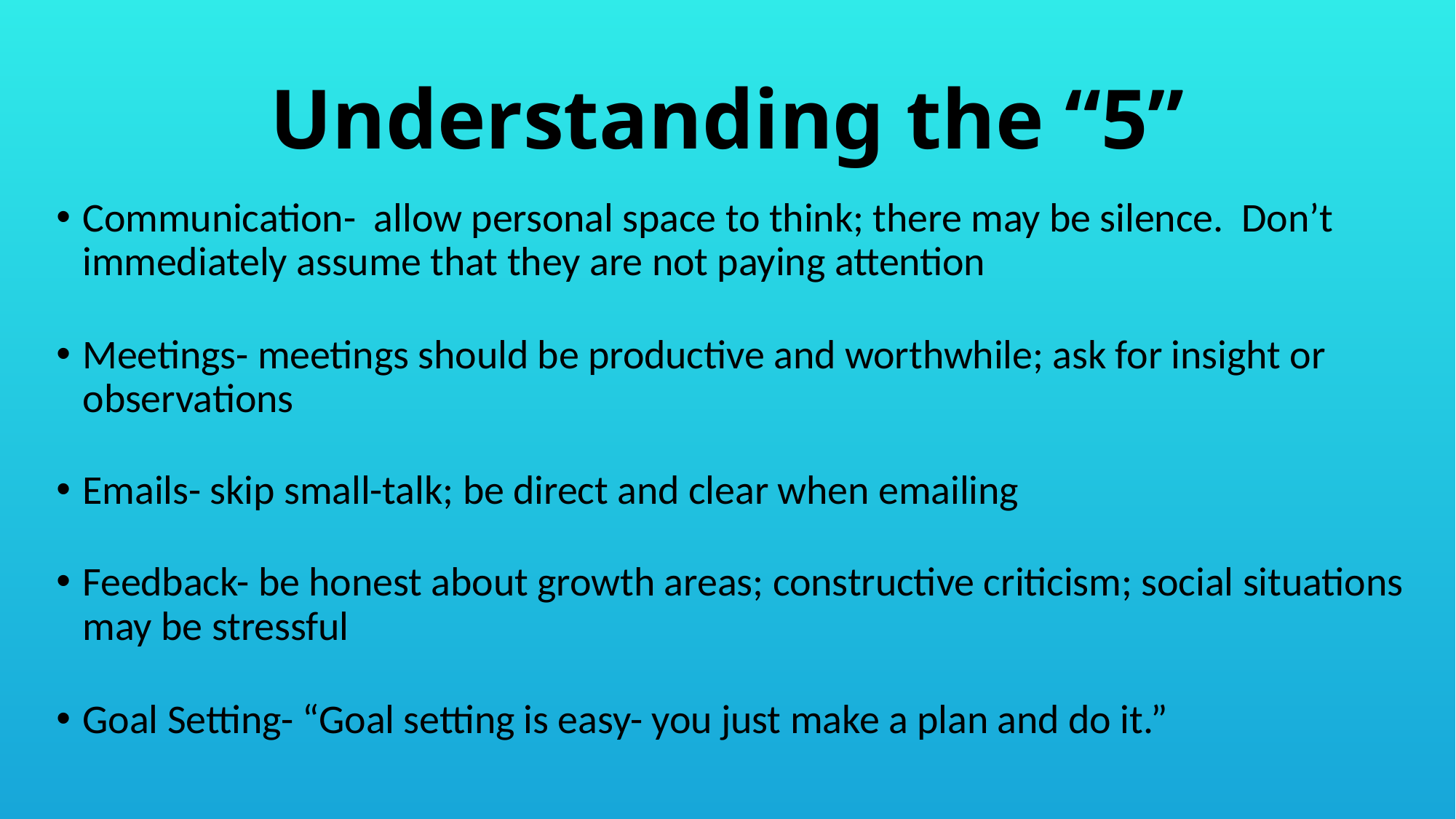

# Understanding the “5”
Communication- allow personal space to think; there may be silence. Don’t immediately assume that they are not paying attention
Meetings- meetings should be productive and worthwhile; ask for insight or observations
Emails- skip small-talk; be direct and clear when emailing
Feedback- be honest about growth areas; constructive criticism; social situations may be stressful
Goal Setting- “Goal setting is easy- you just make a plan and do it.”

## Slide 22
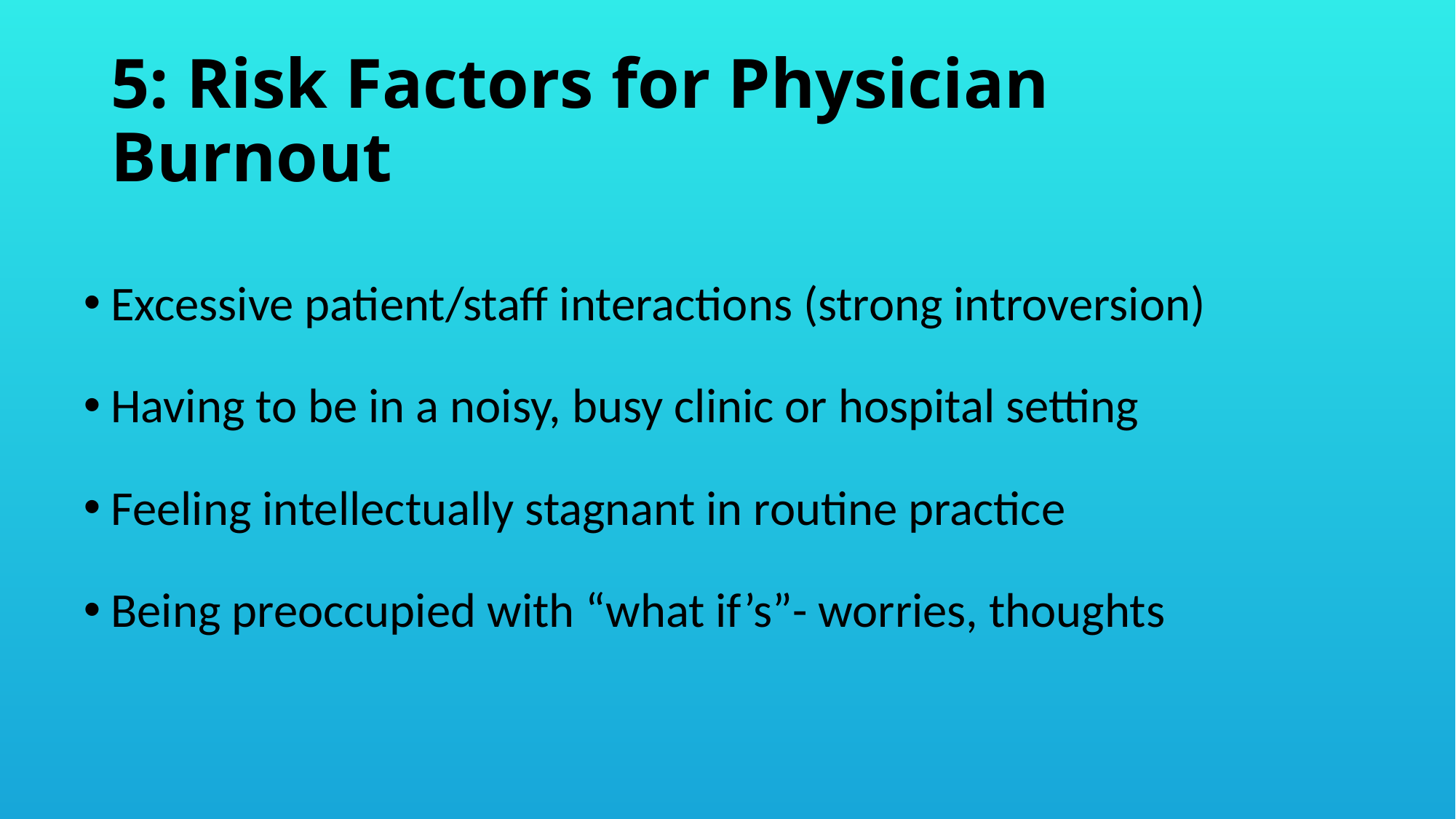

# 5: Risk Factors for Physician Burnout
Excessive patient/staff interactions (strong introversion)
Having to be in a noisy, busy clinic or hospital setting
Feeling intellectually stagnant in routine practice
Being preoccupied with “what if’s”- worries, thoughts

## Slide 23
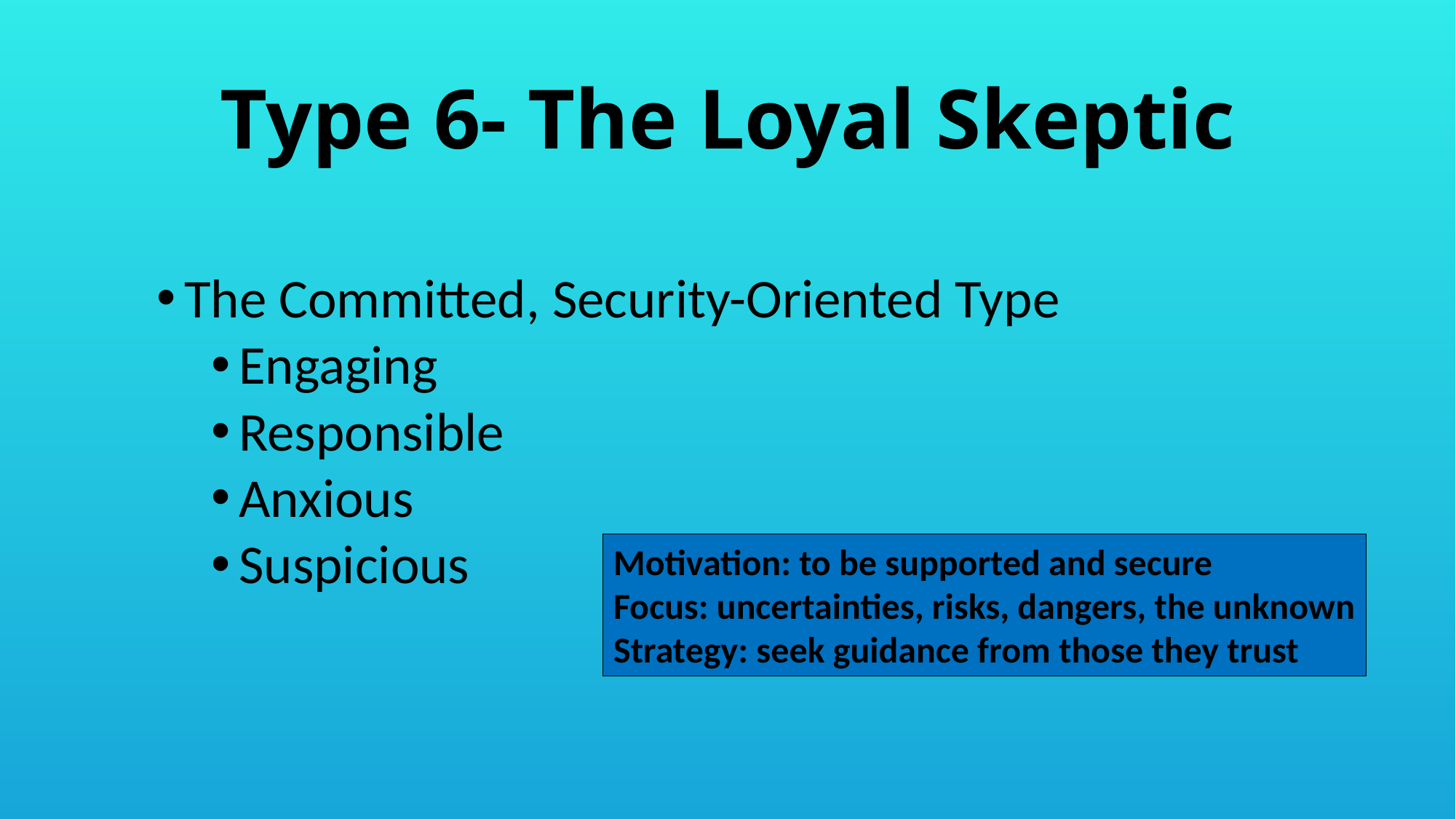

# Type 6- The Loyal Skeptic
The Committed, Security-Oriented Type
Engaging
Responsible
Anxious
Suspicious
Motivation: to be supported and secure
Focus: uncertainties, risks, dangers, the unknown
Strategy: seek guidance from those they trust

## Slide 24
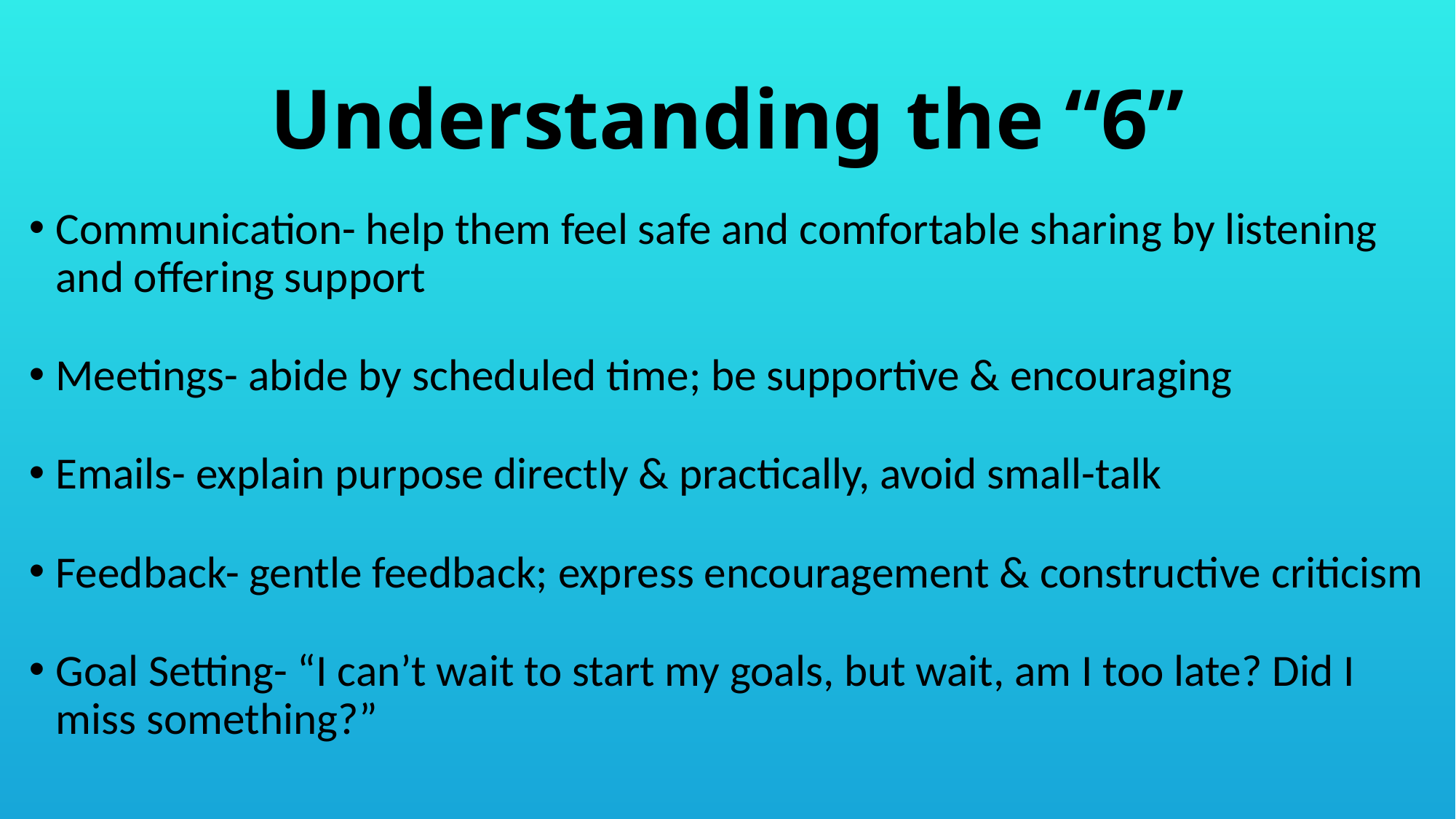

# Understanding the “6”
Communication- help them feel safe and comfortable sharing by listening and offering support
Meetings- abide by scheduled time; be supportive & encouraging
Emails- explain purpose directly & practically, avoid small-talk
Feedback- gentle feedback; express encouragement & constructive criticism
Goal Setting- “I can’t wait to start my goals, but wait, am I too late? Did I miss something?”

## Slide 25
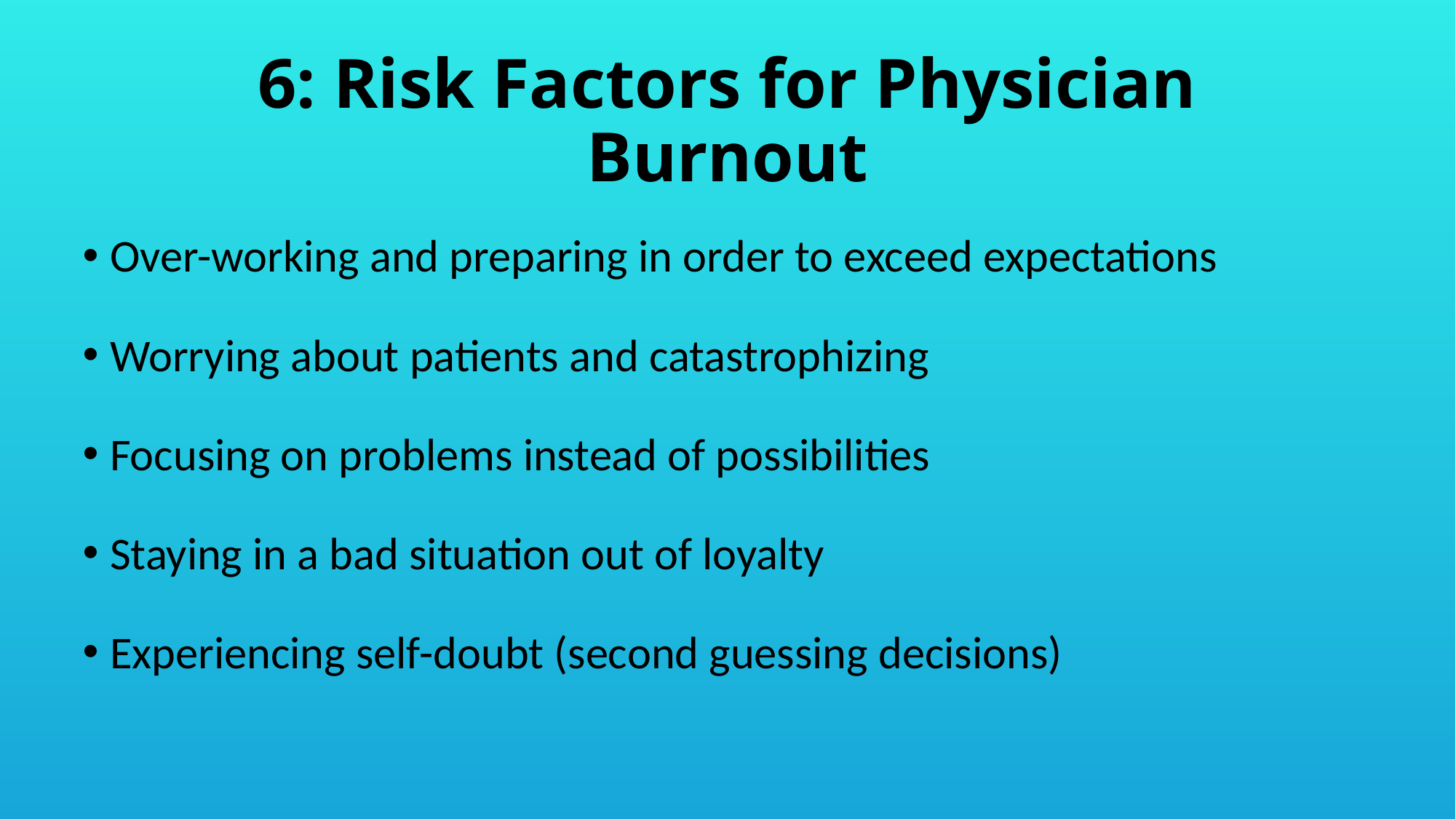

# 6: Risk Factors for Physician Burnout
Over-working and preparing in order to exceed expectations
Worrying about patients and catastrophizing
Focusing on problems instead of possibilities
Staying in a bad situation out of loyalty
Experiencing self-doubt (second guessing decisions)

## Slide 26
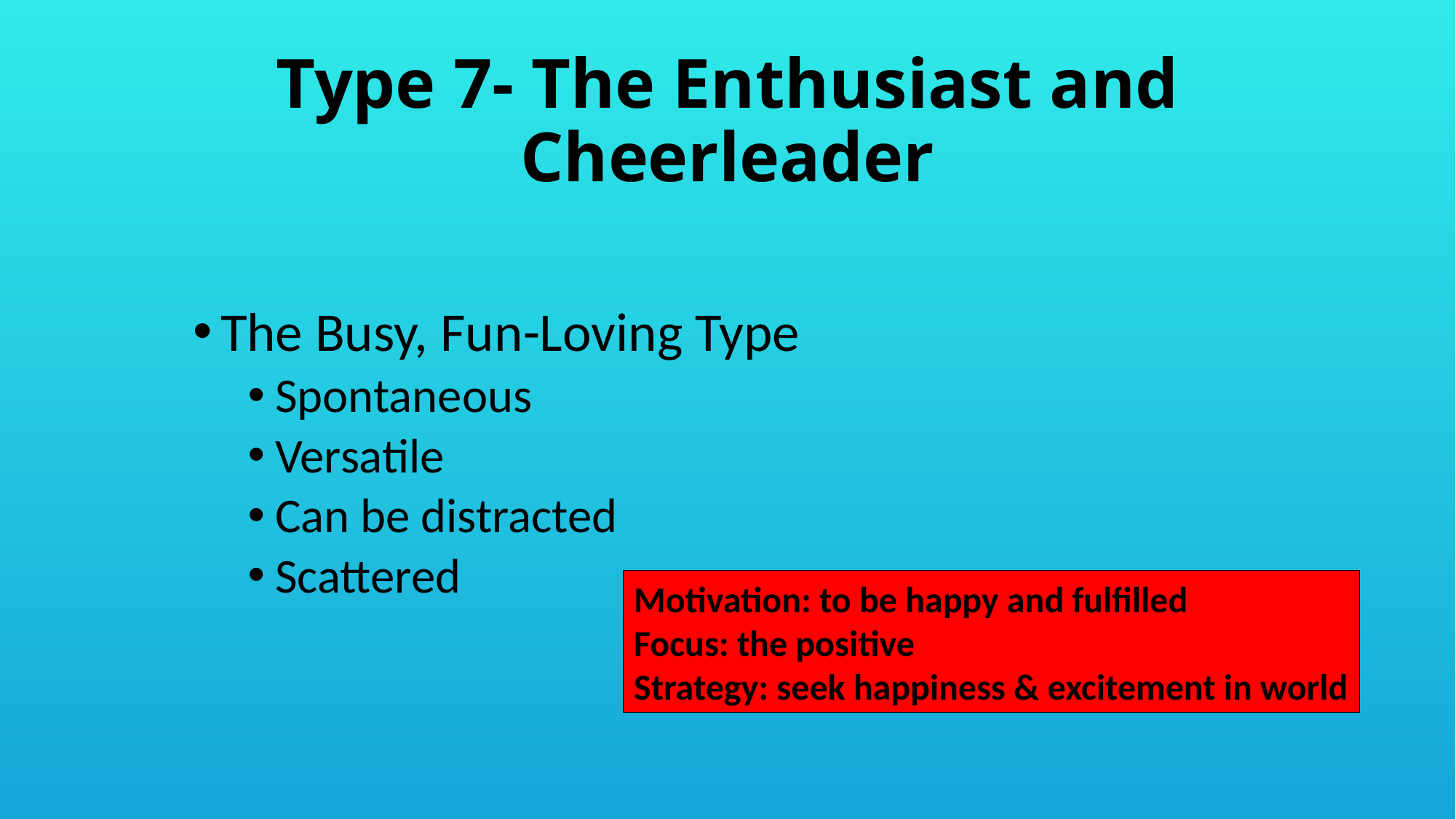

# Type 7- The Enthusiast and Cheerleader
The Busy, Fun-Loving Type
Spontaneous
Versatile
Can be distracted
Scattered
Motivation: to be happy and fulfilled
Focus: the positive
Strategy: seek happiness & excitement in world

## Slide 27
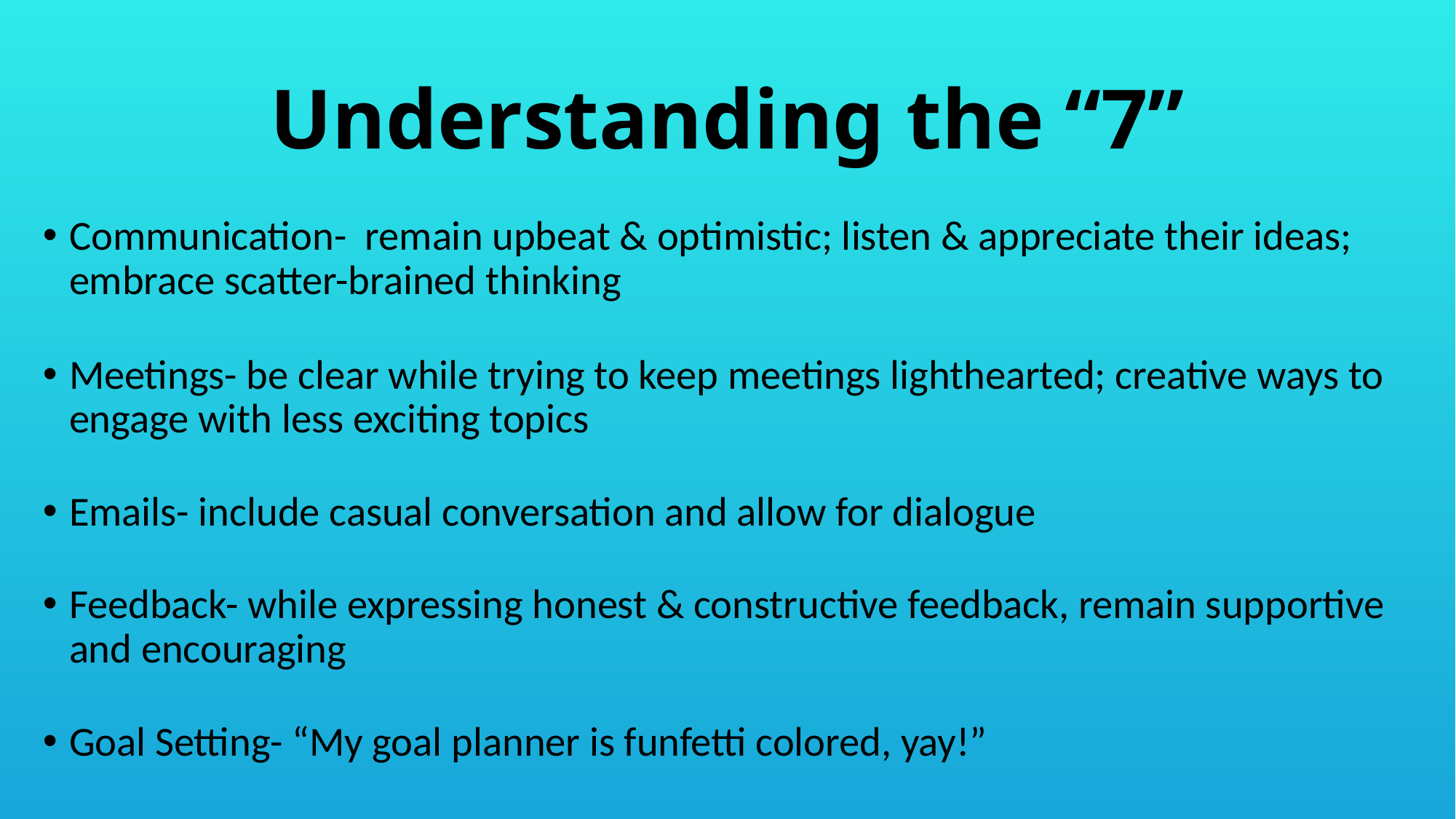

# Understanding the “7”
Communication- remain upbeat & optimistic; listen & appreciate their ideas; embrace scatter-brained thinking
Meetings- be clear while trying to keep meetings lighthearted; creative ways to engage with less exciting topics
Emails- include casual conversation and allow for dialogue
Feedback- while expressing honest & constructive feedback, remain supportive and encouraging
Goal Setting- “My goal planner is funfetti colored, yay!”

## Slide 28
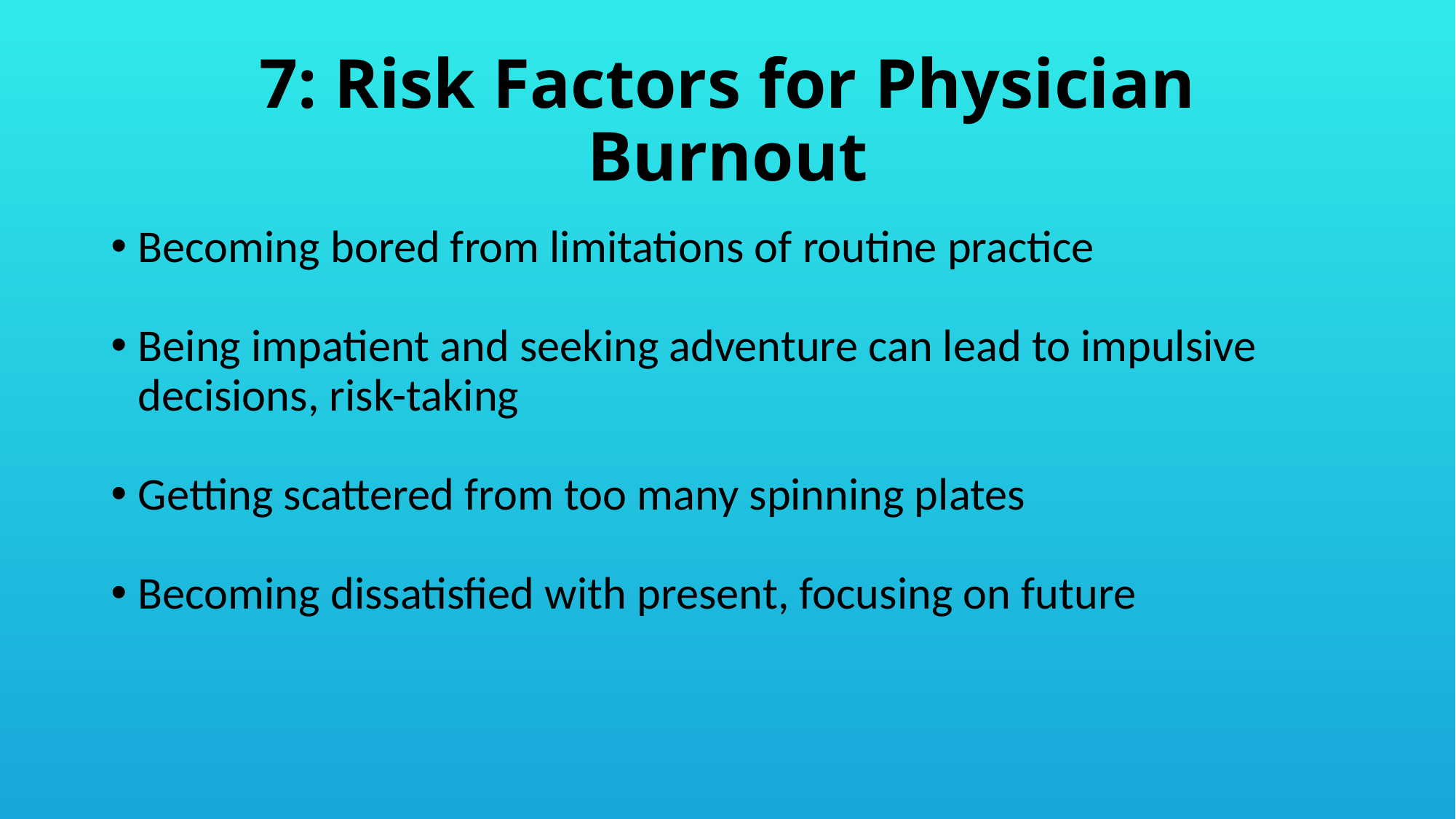

# 7: Risk Factors for Physician Burnout
Becoming bored from limitations of routine practice
Being impatient and seeking adventure can lead to impulsive decisions, risk-taking
Getting scattered from too many spinning plates
Becoming dissatisfied with present, focusing on future

## Slide 29
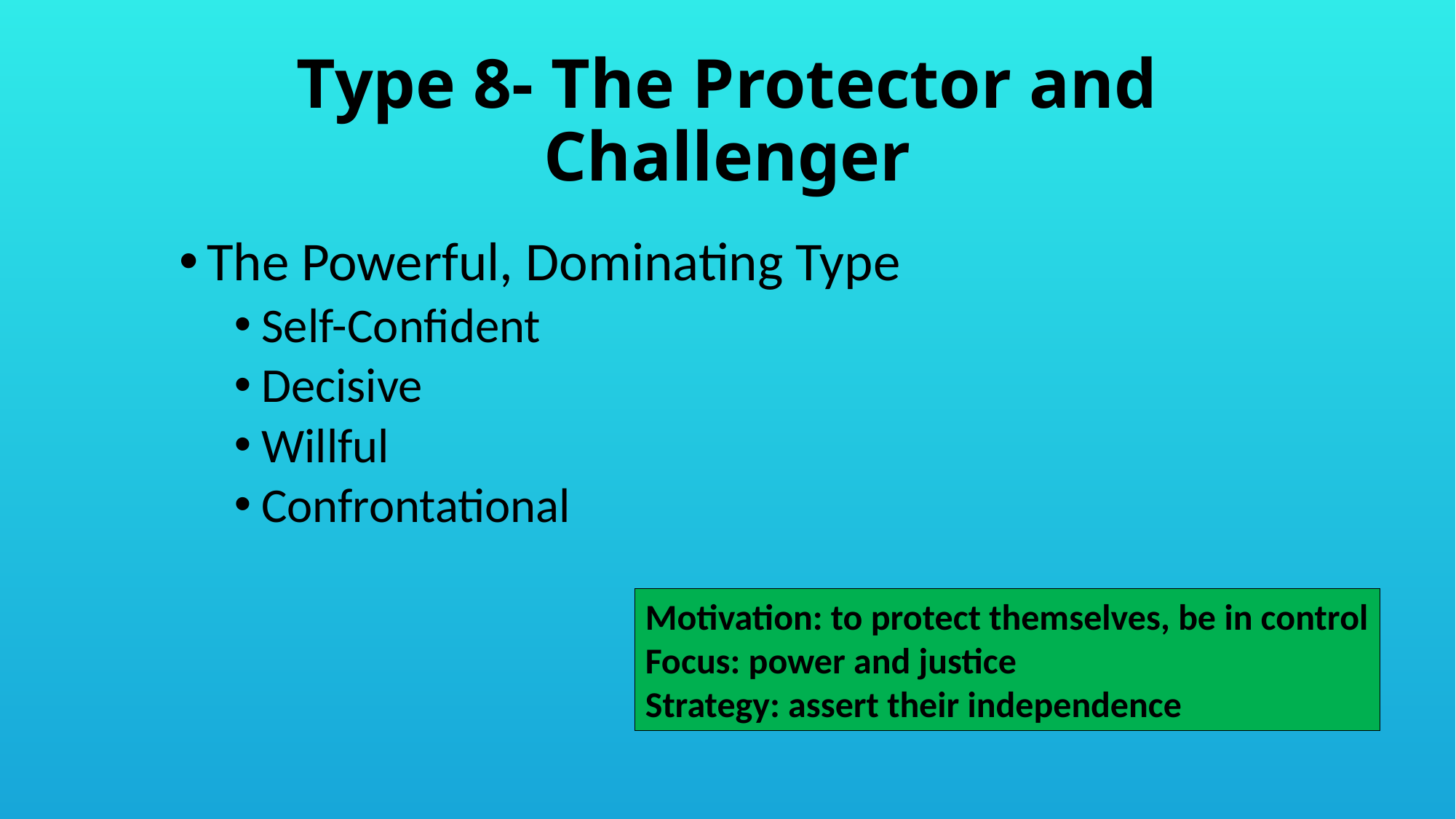

# Type 8- The Protector and Challenger
The Powerful, Dominating Type
Self-Confident
Decisive
Willful
Confrontational
Motivation: to protect themselves, be in control
Focus: power and justice
Strategy: assert their independence

## Slide 30
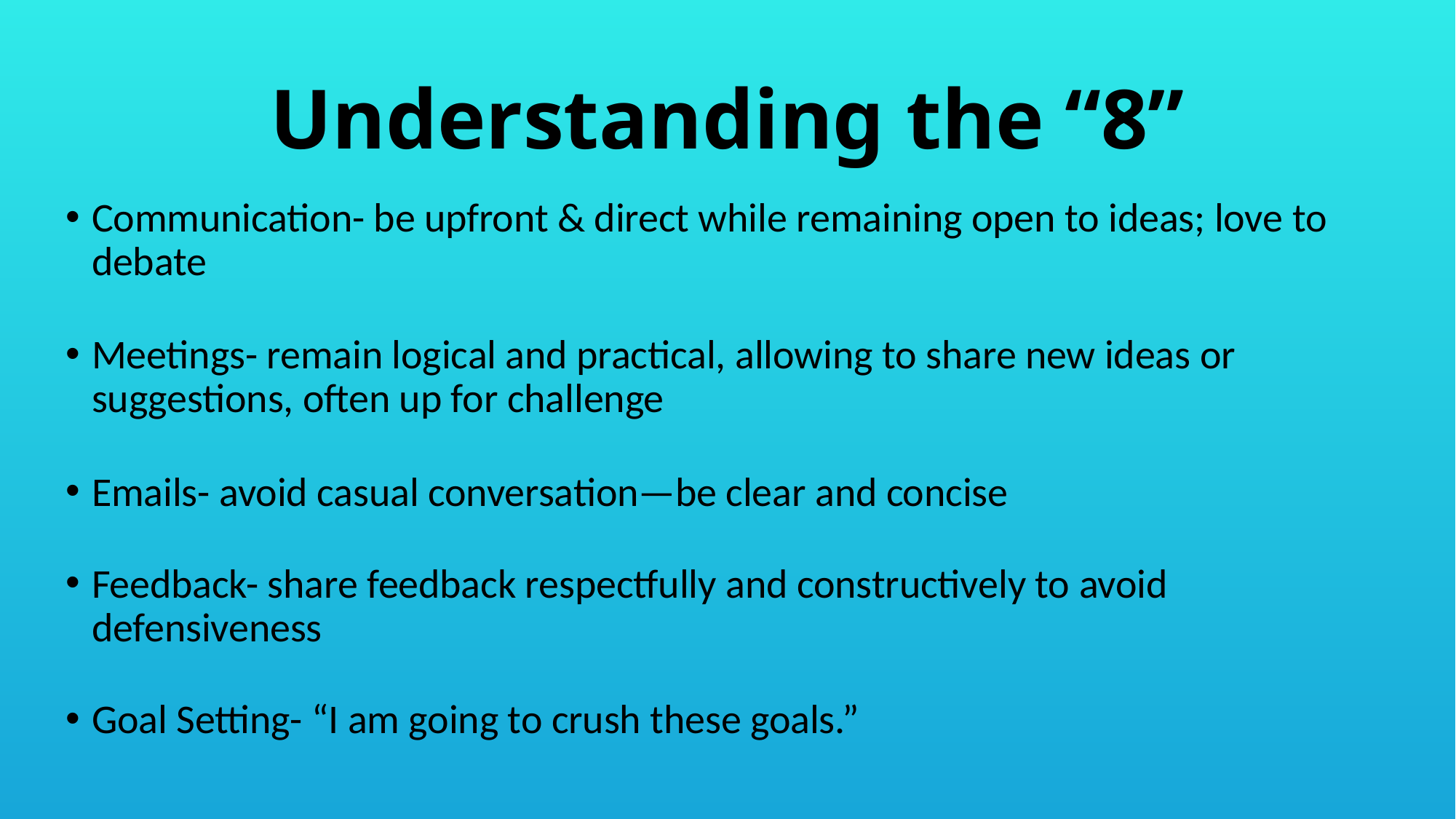

# Understanding the “8”
Communication- be upfront & direct while remaining open to ideas; love to debate
Meetings- remain logical and practical, allowing to share new ideas or suggestions, often up for challenge
Emails- avoid casual conversation—be clear and concise
Feedback- share feedback respectfully and constructively to avoid defensiveness
Goal Setting- “I am going to crush these goals.”

## Slide 31
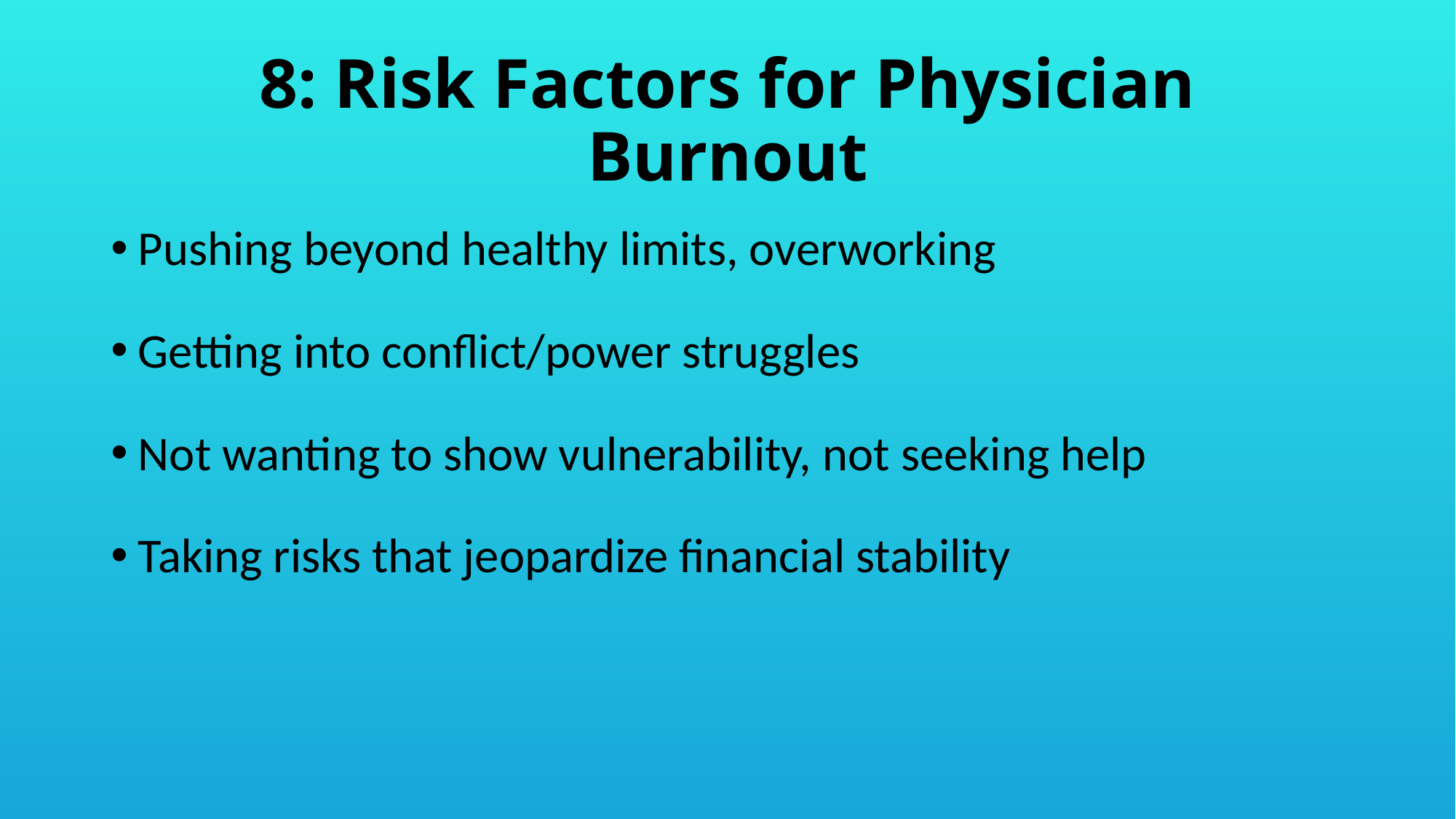

# 8: Risk Factors for Physician Burnout
Pushing beyond healthy limits, overworking
Getting into conflict/power struggles
Not wanting to show vulnerability, not seeking help
Taking risks that jeopardize financial stability

## Slide 32
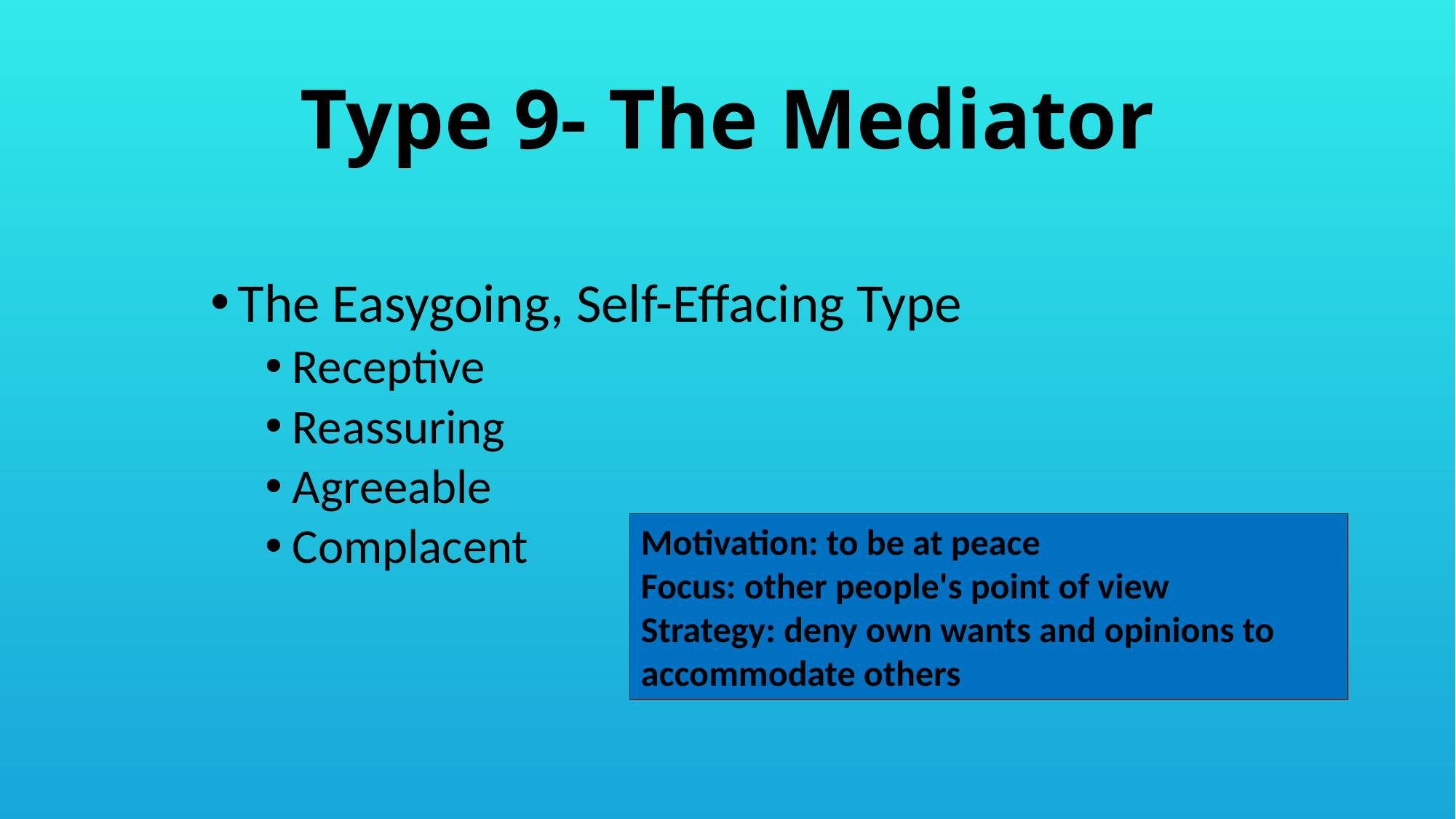

# Type 9- The Mediator
The Easygoing, Self-Effacing Type
Receptive
Reassuring
Agreeable
Complacent
Motivation: to be at peace
Focus: other people's point of view
Strategy: deny own wants and opinions to accommodate others

## Slide 33
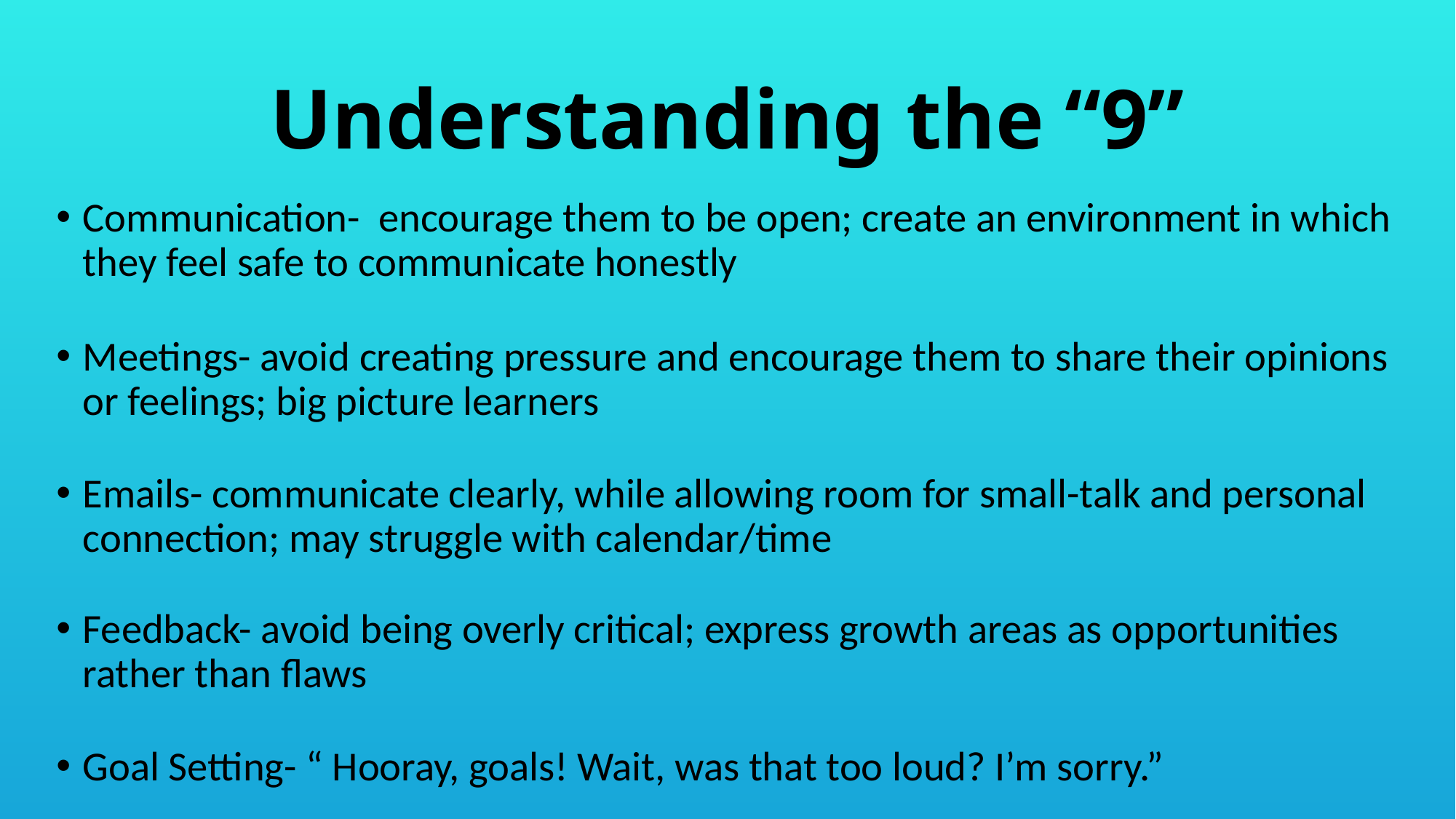

# Understanding the “9”
Communication- encourage them to be open; create an environment in which they feel safe to communicate honestly
Meetings- avoid creating pressure and encourage them to share their opinions or feelings; big picture learners
Emails- communicate clearly, while allowing room for small-talk and personal connection; may struggle with calendar/time
Feedback- avoid being overly critical; express growth areas as opportunities rather than flaws
Goal Setting- “ Hooray, goals! Wait, was that too loud? I’m sorry.”

## Slide 34
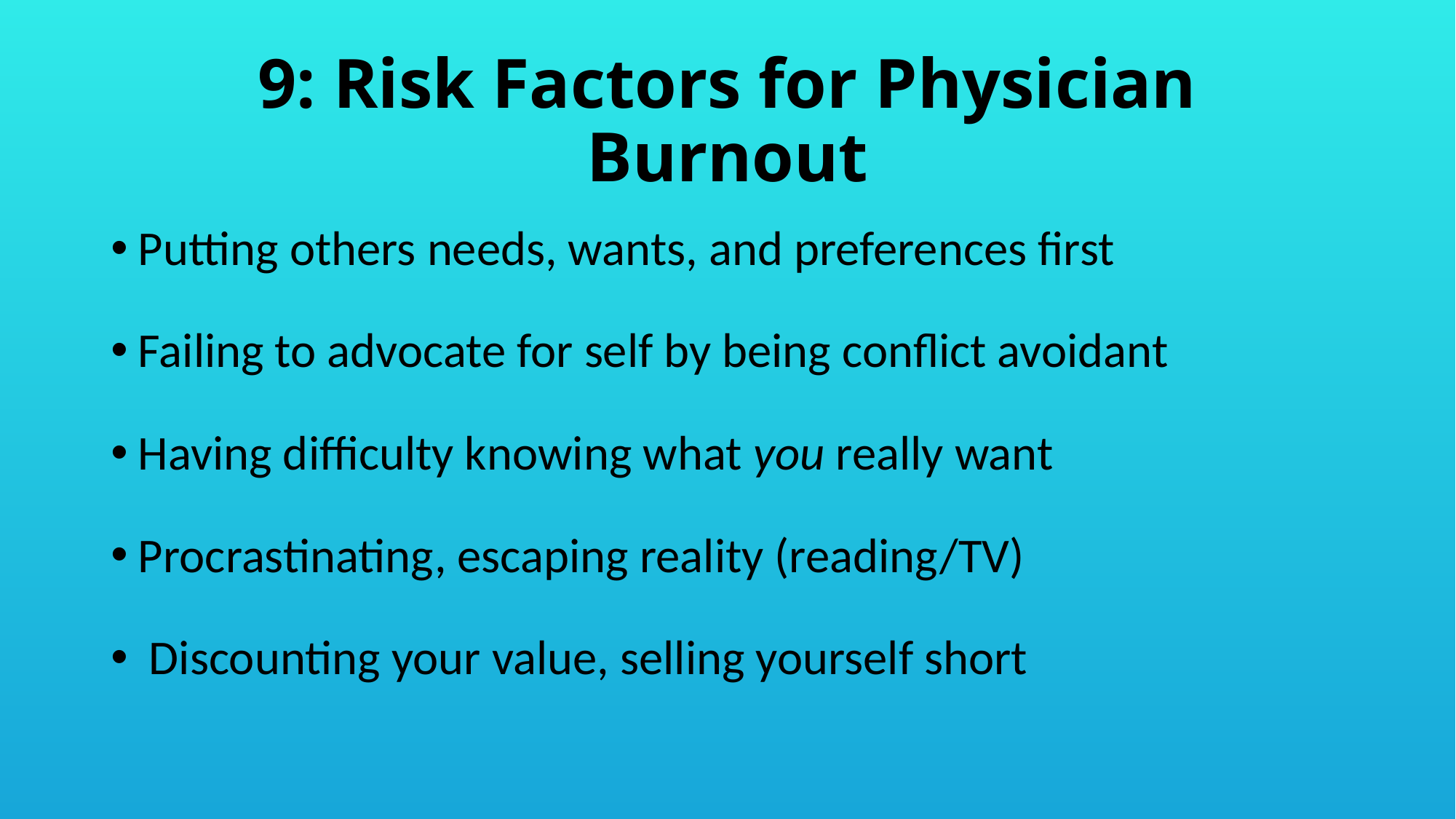

# 9: Risk Factors for Physician Burnout
Putting others needs, wants, and preferences first
Failing to advocate for self by being conflict avoidant
Having difficulty knowing what you really want
Procrastinating, escaping reality (reading/TV)
 Discounting your value, selling yourself short

## Slide 35
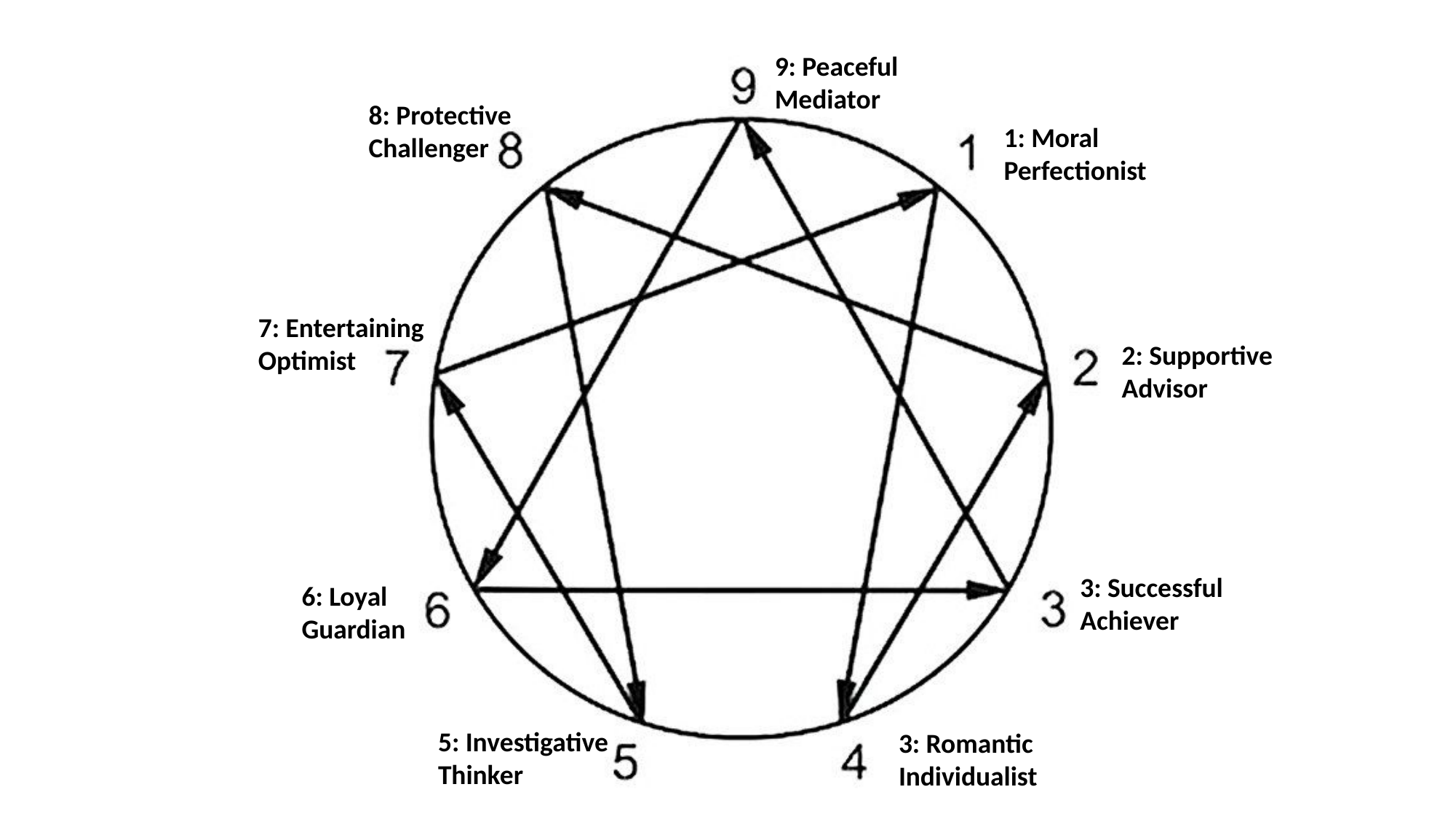

9: Peaceful
Mediator
8: Protective
Challenger
1: Moral
Perfectionist
7: Entertaining
Optimist
2: Supportive
Advisor
3: Successful
Achiever
6: Loyal
Guardian
5: Investigative
Thinker
3: Romantic
Individualist
